# Photolytic mechanisms of hydroxylamine†

**DOI:** 10.1039/c9ra10956k

**Published:** 2020-02-26

**Authors:** Jittima Thisuwan, Phorntep Promma, Kritsana Sagarik

**Affiliations:** Division of Science, Faculty of Education, Nakhon Phanom University Nakhon Phanom 48000 Thailand; School of Chemistry, Institute of Science, Suranaree University of Technology Nakhon Ratchasima 30000 Thailand kritsana@sut.ac.th +66 44 224635 +66 44 224635

## Abstract

The photodissociation of small molecules has been extensively studied because of the increase in environmental problems related to the atmosphere of the Earth. In this work, the photodissociation mechanisms of hydroxylamine (NH_2_OH) as a model molecule in its lowest singlet-excited (S_1_) state were systematically studied using the complete active space second-order perturbation theory (CASPT2) and transition state theory (TST). In particular, this study focused on nonradiative relaxation processes that convert the S_0_ → S_1_ excited-state molecule to its products in their respective electronic ground states. The potential energy curves obtained from relaxed scans suggest that O–H dissociation is the preferred process in the S_1_ state. For the N–O and N–H dissociation pathways, thermally excited precursors were hypothesized to form in the S_0_ state to circumvent O–H dissociation. Thus, S_0_ → S_1_ vertical excitations lead to transition structures in the S_1_ state, which fragment to their respective electronic-ground-state products. The thermodynamic and kinetic results confirmed the precursor hypothesis, showing that the exothermic energy caused by the formation of HNO and H_2_ is sufficient to generate such precursors in the S_0_ state. Additionally, the TST confirmed that unimolecular isomerization–dissociation is a two-step process that generates products effectively by direct photolysis of the corresponding covalent bonds. In particular, the process consists of O–H bond dissociation, followed by spontaneous isomerization and formation of H_2_ in its electronic ground state, resulting in the high quantum yield observed in the UV absorption experiments in the preferential formation of HNO and H_2_. The configuration interaction coefficients of the characteristic structures on the potential energy curves revealed considerable changes in the multiconfigurational character of the wavefunctions, especially for the transition structures. These are characterized by the development of Rydberg orbitals, being produced at the intersection of the S_0_ and S_1_ states. The present study highlights the effects of thermal selectivity and the multiconfigurational character of the wavefunctions on photodissociation. Because detailed information on the photolytic mechanisms of isolated NH_2_OH is limited both theoretically and experimentally, these results provide fundamental insight into unimolecular photodissociation, posing ground for future studies on related systems.

## Abstract 2

Photolysis of NH_2_OH characterized by Rydberg orbitals resulting from significant changes in multiconfigurational characters and thermal excitation in the electronic ground state.

## Graphical abstract

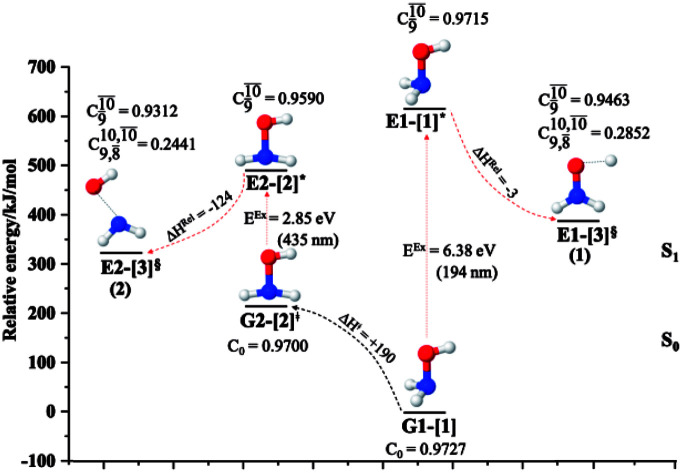

## Introduction

The photodissociation of small molecules has been extensively studied both theoretically and experimentally because environmental problems related to the atmosphere of the Earth are increasing.^1^ Because it has O–H and N–H groups, as well as lone-pair electrons, hydroxylamine (NH_2_OH) has often been employed as a prototypical molecule in mechanistic studies of gas-phase photodissociation processes.^2–7^ For isolated NH_2_OH, two types of unimolecular photodissociation mechanisms have been reported: (i) direct photolysis of the O–H, N–O, and N–H covalent bonds,^3^ which can generate the nitroxyl (NH_2_O), hydroxylamino (NHOH), amino (NH_2_), hydroxyl (OH) radical groups, and hydrogen (H); (ii) intramolecular isomerization/dissociation, which can produce, *e.g.*, nitrosyl hydride (HNO)^8–10^ and ammonia oxide (NH_3_O).^11–14^

In the direct photolysis pathway, ultraviolet (UV) absorption experiments have shown that the H-atom channel, in which two H atoms are generated with a quantum efficiency greater than one (1.7), is the preferred process at an absorption wavelength of 193 nm. In this pathway, N–O dissociation is a minor process, with a quantum efficiency of less than 0.1.^5^ Instead, photolysis by UV absorption at 240 nm leads mainly to the dissociation of N–O and formation of NH_2_ and OH in their electronic ground states.^7^ Thus, though the O–H dissociation was first proposed, both O–H and N–O dissociation have been reported as primary processes (representing 60% and 40%, respectively) in the direct photolysis of NH_2_OH vapor at 298 K, because of the possible thermal decomposition.^3^

Analysis of the H-atom Doppler profiles^5^ suggested that the only energetically accessible path to generate two H atoms is

1 NH_2_OH + *hν* → H + HNO + H,

and that the two H atoms are produced in two stepwise-decay processes, which are

2 NH_2_OH + *hν* → H + NH_2_O → H + HNO + H

and

3 NH_2_OH + *hν* → H + NHOH → H + HNO + H.

Although the final products of eqn (2) and (3) are identical, the intermediates are different. Therefore, it is necessary to determine which covalent bond, O–H or N–H, dissociates first. Limited evidence suggests that the N–H dissociation of eqn (3) occurs first.^5^

To study the photodissociation of NH_2_OH, *ab initio* calculations have been previously performed on low-lying singlet states using the complete active space self-consistent field (CASSCF) method.^7^ The potential energy curves obtained from the freeze-scan method, in which the remaining coordinates were fixed at their MP2/6-31G** equilibrium values in the electronic ground state, showed that excitations from the two lowest-lying singlet states (*n*_orb_ = 8 and 9) are possible and can lead to fragmentations through the H-atom, NH_2_, and OH channels. It was concluded that these two dissociation processes result from excitations with different wavelengths rather than different excitation mechanisms.^7^ This is consistent with other *ab initio* calculations based on the coupled electron pair approximation, which showed that the two highest occupied orbitals, 2a′′ and 7a′, are associated with the 2p lone-pair orbitals of the O and N atoms, respectively, with an energy difference of less than 1 eV.^15^

The end product of NH_2_OH photolysis at 193 nm (6.42 eV)^5^ is HNO, which is an important intermediate in the formation of NO by combustion^16^ and by the catalytic decomposition of ozone (O_3_) in the stratosphere and reaction with HNOH.^17^ HNO is a reactive radical with a rather long lifetime (0.1 s),^18^ and it has been studied extensively both experimentally and theoretically.^8^ Although computational studies have suggested the formation of triplet HNO (^3^HNO), the bent structure in the singlet state (^1^HNO) was concluded to be the most stable,^19^ with a singlet–triplet energy gap of 77 kJ mol^−1^.^20^ Using thermodynamic data, the threshold wavelength (*λ*_thres_) for the formation of HNO and H_2_ after excitation of NH_2_OH by 193 nm-UV radiation was predicted to be 891 nm (1.39 eV).^5^

The interconversion between NH_2_OH and NH_3_O is a prototypical model for unimolecular chemical transformations (*i.e.*, isomerization).^11^ Although the energy barrier associated with intramolecular hydrogen/proton transfer from the O atom to N is rather high in the electronic ground state of this system (∼234 kJ mol^−1^),^11^ mass spectroscopic experiments and *ab initio* calculations confirmed the existence of neutral NH_3_O in the gas phase,^12^ and structure–reactivity analysis of the equilibrium constants suggested that ∼20% of aqueous NH_2_OH solution is composed by NH_3_O.^13^

**Fig. 1:**
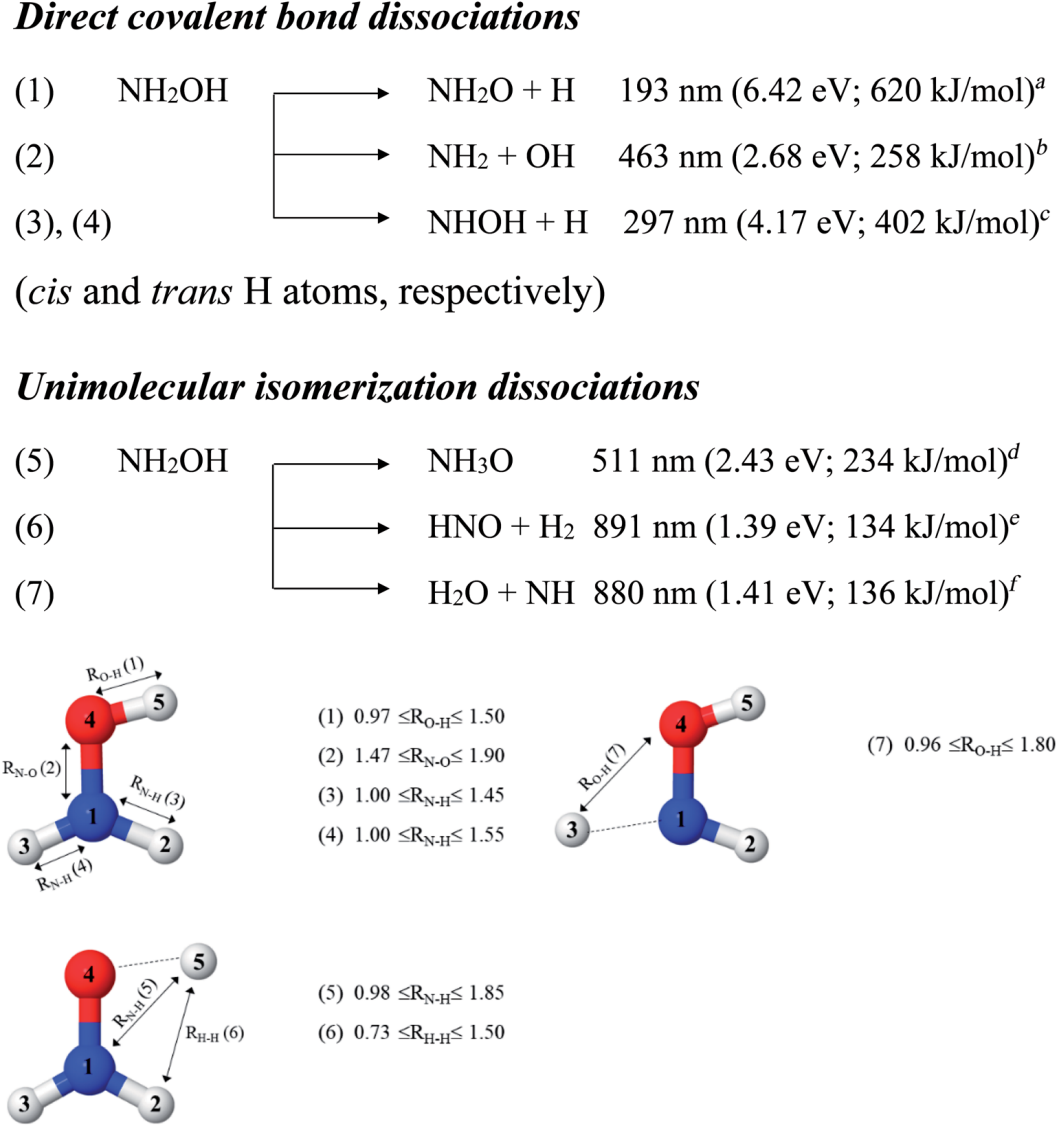
Direct covalent bond dissociations and unimolecular-isomerization dissociations for NH_2_OH in the gas phase suggested based on experiments and theories. Double sided arrows represent the degrees of freedom used in the calculations of the potential energy curves. Distances are in Å. (…) = dissociation channel. ^*a*^UV absorption in ref. 5; ^*b*^the value reported in Table II of ref. 7 based on the analysis of the data in ref. 41; ^*c*^threshold energy for the photodissociation of NH_3_ into NH and H_2_ from photoemission in ref. 42; ^*d*^*ab initio* calculations in the electronic ground state in ref. 11; ^*e*^thermodynamic data in ref. 5; ^*f*^thermodynamic data and *ab initio* calculations in ref. 43.

In this work, the photolytic mechanisms of a single NH_2_OH molecule in the lowest singlet-excited (S_1_) state were studied using *ab initio* calculations through the complete active space second-order perturbation theory (CASPT2) with the aug-cc-pVDZ basis set. Different from previous ones, this study focuses on nonradiative relaxation processes that convert the excited-state molecule to its electronic-ground-state products and on the effects of thermal energy on photodissociation. The structures and energetics of the precursors, and the transition structures of the seven dissociation channels, shown in Fig. 1, were studied in detail using the S_0_ and S_1_ potential energy curves obtained from CASPT2 and relaxed scans. To determine the contributions of the different electronic states to photodissociation, we analyzed the configuration interaction (CI) coefficients corresponding to the multiconfigurational character of each structure on the potential energy curves. Because of the limited theoretical and experimental information, the role of thermal energy in the photolytic mechanisms was discussed using the transition state theory (TST),^21,22^ considering the 200–1200 K temperature range.

## Computational methods

### *Ab initio* calculations

Because the photodissociation of NH_2_OH involves fragmentation and formation of covalent bonds, closed- and open-shell configurations must be considered.^23^ To account for the multiconfigurational character of NH_2_OH photolysis, *ab initio* calculations were performed using CASPT2, which is a widely recognized method for excited-state calculations.^24^ It should be mentioned that although the multistate complete-active-space second-order perturbation (MS-CASPT2) method is more accurate, MS-CASPT2 calculations are computer intensive and therefore applicable only to small systems. In this work, because the energy gradients with respect to degrees of freedom and Hessian had to be computed extensively, the CASPT2 method was employed to optimize the computational resources.

The electronic ground state of NH_2_OH in its equilibrium structure with *C*_s_ symmetry is represented by (1a′)^2^(2a′)^2^(3a′)^2^(4a′)^2^(1a′′)^2^(5a′)^2^(6a′)^2^(2a′′)^2^(7a′)^2^. The active space was defined by assigning ten electrons (*n* = 10) to nine active orbitals (*m* = 9), and hereafter abbreviated as the (10,9) active space. The remaining electrons were assigned to four doubly occupied orbitals (close = 4). For NH_2_OH, CASPT2(10,9) calculations involved 5292 CASSCF reference wavefunctions. The aug-cc-pVDZ basis set was satisfactorily used to optimize computational resources. Indeed, augmented basis sets with diffuse functions are reportedly suitable for singlet-state calculations,^24^ and in our previous study, CASPT2/aug-cc-pVDZ calculations were shown to yield reasonable potential energy curves and S_0_ → S_1_ vertical excitation energies for water clusters.^25^

The *ab initio* CI calculations in the CASSCF framework^7^ revealed that the first two electronic excited states involve excitations of a single electron from the two highest occupied orbitals, 2a′′ (*n*_orb_ = 8) and 7a′ (*n*_orb_ = 9), to the two lowest unoccupied ones, 8a′ (*n*_orb_ = 10) and 9a′ (*n*_orb_ = 11), and that these low-lying excited states possess Rydberg and dissociative-valence character, which results from adiabatic excitation.^26^ Because of this, the S_1_ state was calculated adiabatically. Schematic diagram showing doubly occupied and active spaces used in CASPT2(10,9) calculations and spatial distributions of the orbitals potentially involved in the S_0_ → S_1_ excitation of NH_2_OH are illustrated in Fig. S1.†

Additionally, because previous *ab initio* calculations suggested that the products of photodissociation forming at conical intersections do not necessarily have *C*_s_ symmetry,^26^ and because nonradiative relaxations of the excited structures are our primary interest, the CASPT2(10,9) geometry was optimized with no geometrical constraints (*C*_1_ symmetry). To study the effects of the multiconfigurational wavefunctions in the photolysis of NH_2_OH, the CI coefficients of the equilibrium, transition, and final structures on the potential energy curves were examined. The CASPT2(10,9) calculations were performed using the MOLPRO software package^27,28^ and applying the Werner–Meyer–Knowles nonlinear method in the orbital/state optimization.^29–31^

### Potential energy curves and equilibrium structures

To obtain information on the equilibrium structures and elementary photodissociation steps, the potential energy curves of the direct O–H, N–O, and N–H dissociations were constructed as relaxed scans in the S_1_ state. Here, the structural parameters of the potential energy curves were optimized using the CASPT2(10,9) and quadratic steepest descent (QSD) methods,^32^ and the same geometries were used to calculate the energies of the S_0_ potential energy curves. All degrees of freedom used in these *ab initio* calculations are included in Fig. 1.

Because our preliminary CASPT2(10,9) results showed that the O–H dissociation of channel (1) occurs preferentially along a purely repulsive potential energy curve in the S_1_ state, the S_1_ potential energy curves for the N–O and N–H dissociation of channels (2)–(4) were constructed by constraining the O–H(5) distance at the equilibrium value of the ground (S_0_) state (*R*_O–H(5)_ = 0.97 Å). These calculated potential energy curves were used to characterize the potential precursors in their electronic ground state outside the Franck–Condon region of the equilibrium structure. From these, the S_0_ → S_1_ vertical excitations could provide the transition and final structures in their respective ground state.

Because mass spectroscopy measurements confirmed the existence of neutral NH_3_O in the gas phase,^12^ and because intramolecular isomerization is one of the most common radical reactions in electronic excited states, the unimolecular isomerization potential energy curve^11^ for the formation of ammonia oxide (NH_2_OH → NH_3_O) of channel (5) was constructed in the S_1_ state by transferring the dissociated H(5) atom of channel (1) to the N atom (Fig. 1). Similarly, because HNO and H_2_ are the dominant products of photolysis at the lowest absorption wavelength (193 nm),^5^ and because O–H dissociation is the primary process, an intramolecular isomerization that generates HNO and H_2_ (channel (6)) was assumed by transferring the dissociated H atom of channel (1) (H(5)) to one of the H atoms (H(2) or H(3)) of the NH_2_ group (Fig. 1). A high energy barrier caused by intramolecular rearrangement was assumed for the H_2_ generation mechanisms.^7^

Although NH was not detected during UV photolysis at 193 nm,^7^ it is interesting to calculate the transition structures and energetics of the H(2) → O and H(3) → O isomerization of channel (7) and compare them with those of the H(5) → N isomerization of channel (5). Similar to the approach used for N–O and N–H dissociation, the unimolecular isomerization–dissociation process that underlies the formation of NH and H_2_O was simulated by constraining the O–H(5) distance at 0.97 Å and transferring the dissociated H atom of channel (3) or (4) (H(2) or H(3)) to the O atom.

### The effects of thermal energy on photodissociation

Because the potential energy curves obtained by CASPT2(10,9) calculations represent the reaction paths at 0 K, the effects of temperature must be incorporated in the model. The mechanisms represented in Fig. 1 involve covalent bond dissociation and isomerization of a single molecule. Therefore, unimolecular rate constants (*k*) were used, calculating them in the 200–1200 K temperature range according to TST,^21,22^ which can be applied when the energy barrier is higher than the thermal energy (*k*_B_*T*).^33^ Although some of the direct covalent bond dissociations involve transferring the H atom, the S_0_ and S_1_ potential energy curves evidenced that the S_0_ state has a broad energy barrier, whereas S_1_ is barrierless, implying that quantum mechanical tunneling has no significant role. Therefore, the classical (*k*^Class^) and quantized-vibrational (*k*^Q-vib^, which includes the zero-point vibrational energies) rate constants were initially computed. For the one-dimensional energy profile, the classical transition rate constant is expressed as^34^4
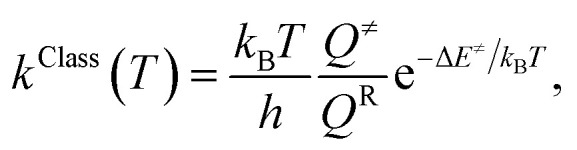
where *Q*^≠^ and *Q*^R^ are the partition functions of the transition and reactant structures, respectively, and Δ*E*^≠^ is the potential energy barrier obtained from the relaxed-scan potential energy curve. *k*_B_ and *h* are the Boltzmann and Planck constants, respectively.

To calculate the rate constant with quantized vibrations, the barrier height obtained with the zero-point vibrational energy (Δ*E*^≠^_ZPE_) is used, and the partition functions are calculated in the harmonic oscillator approximation:5
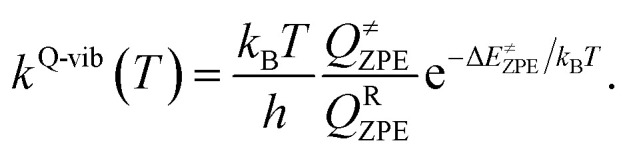
Here, *Q*^≠^_ZPE_ and *Q*^R^_ZPE_ are the partition functions of the transition and reactant structures obtained with respect to their zero-point vibrational energies. Additionally, the crossover temperature (*T*_c_), *i.e.*, the temperature below which the transition states are dominated by quantum mechanical tunneling, was computed as^35,36^6
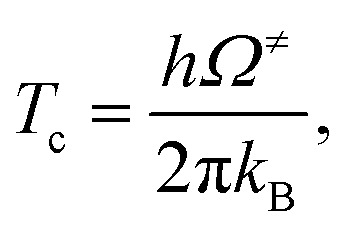
where *Ω*^≠^ is the imaginary frequency of the transition structure. Although the effects of thermal energy are discussed only for the highest temperature (1200 K), *i.e.*, the temperature at which high energy precursors could be populated, the rate constants with quantized vibrations and second-order Wigner correction (*k*^S-Wig^)^35,36^ were calculated to verify the insignificance of quantum mechanical tunneling. Assuming that tunneling occurs at the top of the barrier, the Wigner correction to the rate constant is7
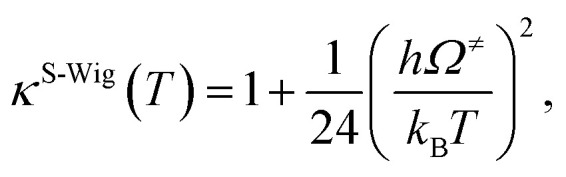
where *κ*^S-Wig^ is the Wigner transmission coefficient, which is 1 in the classical limit (*h* = 0). Then, the Wigner corrected rate constant is

8 *k*^S-Wig^(*T*) = *κ*^S-Wig^(*T*)*k*^Q-vib^(*T*).

Finally, the enthalpy changes (Δ*H*) in the elementary reactions were computed. For the reactions with energy barrier higher than *k*_B_*T*, the linear relationship between ln *k*^Q-vib^(*T*) and 1/*T* was used to calculate the activation enthalpy (Δ*H*^≠^) through the Eyring equation,^34^9
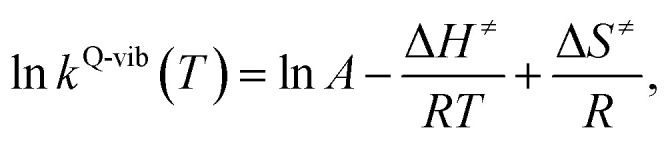
where Δ*S*^≠^ is the activation entropy, and *R* is the gas constant. In these cases, Δ*H*^≠^ was extracted from the slope of the plot. For the elementary reactions with energy barrier lower than *k*_B_*T* (or barrierless), the conventional expression for the relative Gibbs free energy (Δ*G*^Rel^ = Δ*H*^Rel^ − *T*Δ*S*^Rel^) was used to approximate the exothermic enthalpy (Δ*H*^Rel^) as the *y*-intercept of the linear regression of Δ*G*^Rel^ as a function of *T*.

The results confirmed that, for direct covalent bond dissociation at 1200 K, *k*^S-Wig^ is at most 8% higher than *k*^Q-vib^, confirming the applicability of *k*^Q-vib^ to this system. All the transition state calculations were performed using the DL-FIND program^37^ included in the ChemShell package.^38^

## Results and discussion

The characteristic structures of NH_2_OH, identified on the S_0_ and S_1_ potential energy curves, are labeled with a three-character code as Gk-[l], Ek-[l]^≠^, or Ek-[l]*, where G indicates a structure in the S_0_ state, E indicates one in the S_1_ state, and k indicates dissociation channels (1)–(7). Different NH_2_OH structures in the same dissociation channel are labeled [1], [2], *etc.* The *, §, and ≠ symbols denote vertically excited structures, those at the intersection of the S_0_ and S_1_ potential energy curves, and transition structures, respectively. For instance, structures G1-[1]* and E1-[1]* are identical structures (l = 1) computed in the S_0_ (G) and S_1_ (E) states, respectively, involved in channel (1) O–H dissociation (k = 1). Instead, E2-[2]* and E2-[4]^§^ are different structures (l = 2 and 4) on the S_1_ potential energy curve of N–O dissociation (k = 2); they are a vertically excited structure (*) and a structure at the S_0_–S_1_ state intersection (§), respectively.

**Fig. 2:**
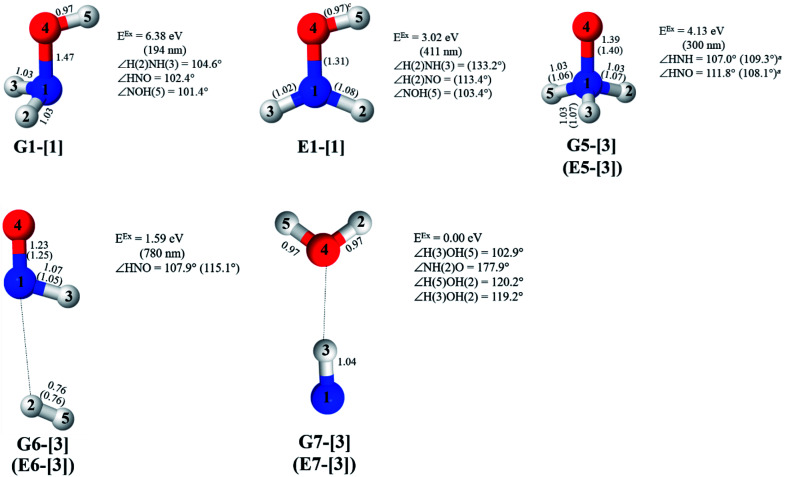
Equilibrium structures of NH_2_OH in the electronic ground (S_0_) and lowest singlet-excited (S_1_) states obtained from CASPT2(10,9) geometry optimizations. Distances and angles are in Å and degree, respectively. (…) are the values obtained in the S_1_ state. The three-character codes are explained in the text. *E*^Ex^ = S_0_ → S_1_ vertical excitation energy. ^*c*^O–H distance constrained in the geometry optimization.

**Fig. 3:**
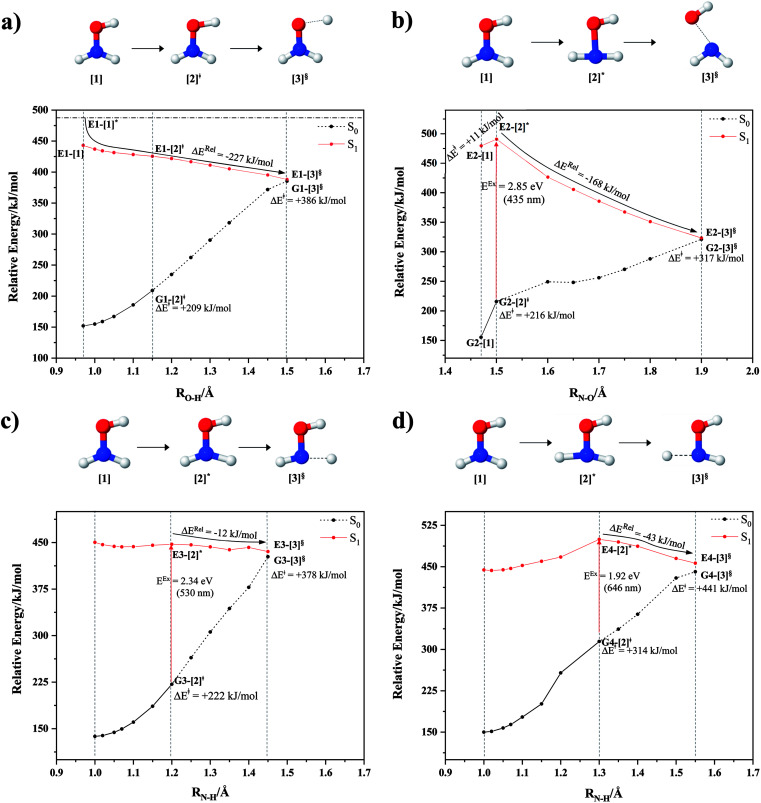
The S_1_ relax-scan potential energy curves for the direct covalent bond dissociations in NH_2_OH obtained from CASPT2(10,9) calculations. The energies on the S_0_ potential energy curves were calculated at the same geometries. The three-character codes are explained in the text. ≠ = transition structure; § = structure at the intersection of the S_0_ and S_1_ potential energy curves; Δ*E*^Rel^ = relative energy with respect to the vertically excited precursor in the S_1_ state; Δ*E*^≠^ = energy barrier with respect to structure G1-[1]; S_0_ and S_1_ = relative energies with respect to the total energy of structure G1-[1], obtained from CASPT2(10,9) calculations in the S_0_ and S_1_ states, respectively. (a–d) O–H, N–O, N–H^*cis*^ and N–H^*trans*^ dissociations, channels (1)–(4), respectively.

**Fig. 4:**
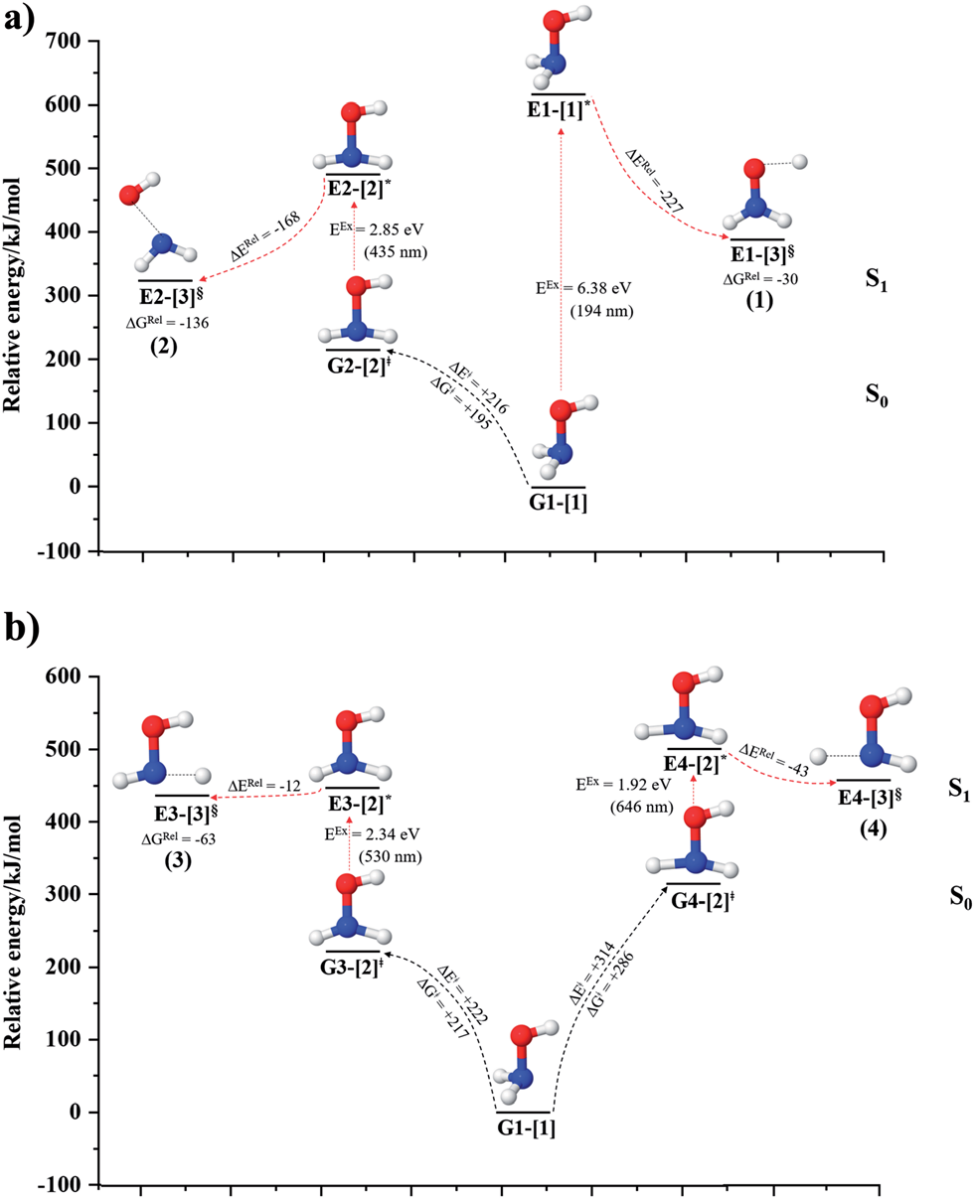
Mechanisms for the direct covalent bond dissociations in NH_2_OH obtained from the analysis of the S_0_ and S_1_ potential energy curves and transition state theories (TST). ≠ = transition structure; § = structure at the intersection of the S_0_ and S_1_ potential energy curves; Δ*E*^≠^ = energy barrier with respect to structure G1-[1]; Δ*G*^≠^ = relative Gibbs free energy barrier with respect to structure G1-[1] at 1200 K; Δ*G*^Rel^ = relative Gibbs free energy with respect to the precursor at 1200 K; *E*^Ex^ = vertical excitation energy; (…) = dissociation channel. (a) Channels (1)–(2). (b) Channel (3)–(4).

**Fig. 5:**
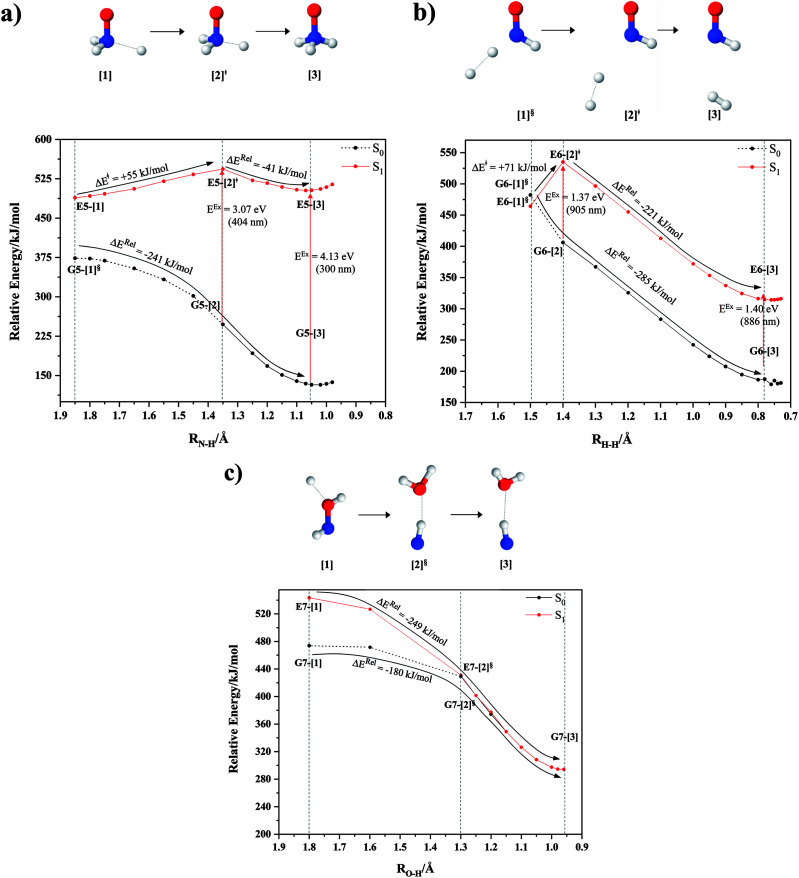
The S_1_ relax-scan potential energy curves for the unimolecular-isomerization dissociations in NH_2_OH obtained from CASPT2(10,9) calculations. The energies on the S_0_ potential energy curves were calculated at the same geometries. The three-character codes are explained in the text. ≠ = transition structure; § = structure at the intersection of the S_0_ and S_1_ potential energy curves; Δ*E*^Rel^ = relative energy with respect to precursor or transition structure; Δ*E*^≠^ = energy barrier with respect to precursor; S_0_ and S_1_ = relative energies with respect to the total energy of structure G1-[1], obtained from CASPT2(10,9) calculations in the S_0_ and S_1_ states, respectively. (a–c) Unimolecular-isomerization dissociations in channels (5)–(7), respectively.

**Fig. 6:**
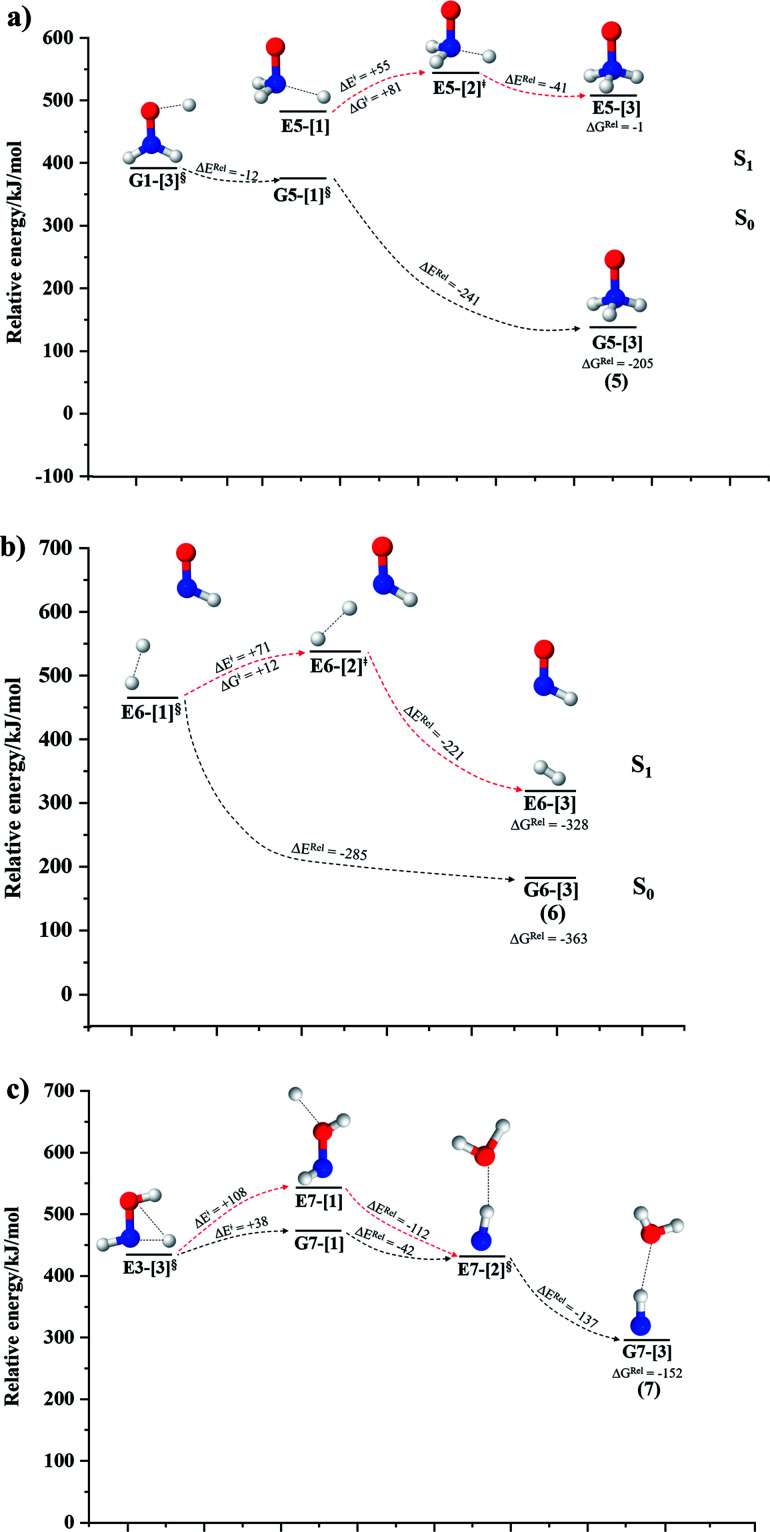
Mechanisms for the unimolecular-isomerization dissociations in NH_2_OH obtained from the analysis of the S_0_ and S_1_ potential energy curves and transition state theories (TST). ≠ = transition structure; § = structure at the intersection of the S_0_ and S_1_ potential energy curves; Δ*E*^≠^ = energy barrier with respect to precursor; Δ*G*^≠^ = relative Gibbs free energy barrier with respect to precursor at 1200 K; Δ*G*^Rel^ = relative Gibbs free energy with respect to the precursor at 1200 K; (…) = dissociation channel. (a–c) Channels (5)–(7), respectively.

The equilibrium structures of NH_2_OH in the electronic ground (S_0_) and lowest singlet-excited (S_1_) states, obtained from CASPT2(10,9) geometry optimizations, are shown in Fig. 2. The relax-scan potential energy curves and proposed mechanisms for the direct covalent bond dissociations are shown in Fig. 3 and 4, respectively. The relax-scan potential energy curves and proposed mechanisms for the unimolecular-isomerization dissociations are illustrated in Fig. 5 and 6, respectively. The calculated CI coefficients are reported in Tables 1 and S1–S7 of the ESI.† Note that *Ψ*_0_ and C_0_ indicate the electronic ground state, *Ψ*^r^_a_ and C^r^_a_ indicate the a → r singly excited state (S-type), and *Ψ*^r,s^_a,b_ and C^r,s^_a,b_ indicate the a → r and b → s doubly excited state (D-type). Indices a/r and b/s correspond to occupied and virtual (or unoccupied) spin orbitals, respectively; the presence or absence of a bar denotes beta (β) or alpha (α) spin orbitals, respectively. The classical and quantum rate constants and relative Gibbs free energies of the elementary reactions are reported in Tables S8–S11.† The vertical excitation energies and corresponding oscillator strengths of characteristic structures are included in Table S12.†

**Table 1:** Equilibrium structures of NH_2_OH and leading CI coefficients obtained from the CASPT2(10,9) method in the S_0_ and S_1_ states. *Ψ*_0_ = electronic ground state; *Ψ*^r^_a_ = a → r singly excited state (S-type); *Ψ*^r,s^_a,b_ = a → r and b → s doubly excited state (D-type); the indices a and b, and r and s label occupied and virtual or unoccupied spin orbitals, respectively; a bar or lack of a bar is to denote beta (β) and alpha (α) spin orbitals, respectively

Structures	S_0_		S_1_	
	Conf.	CI coeff.	Conf.	CI coeff.
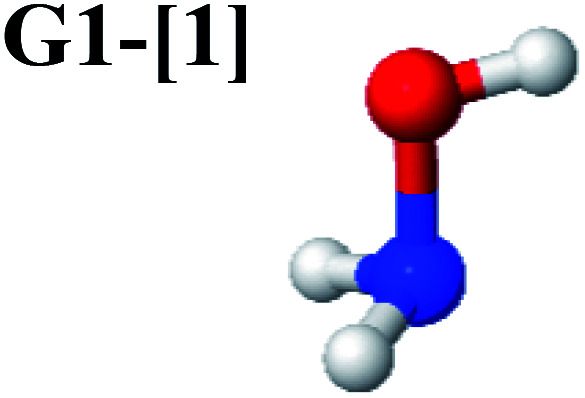	*Ψ*_0_	0.9727	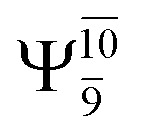	0.9658
	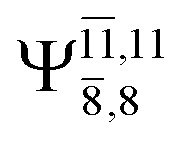	0.1174	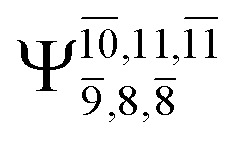	0.1207
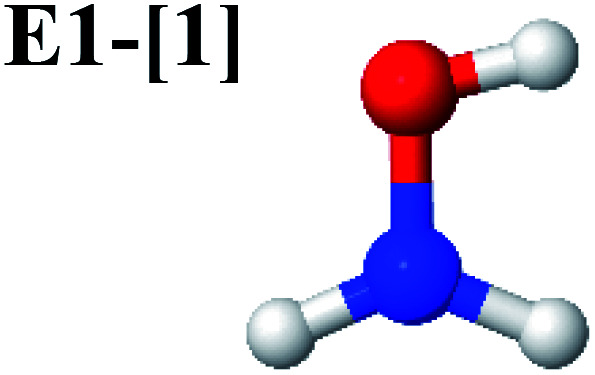	*Ψ*_0_	0.9789	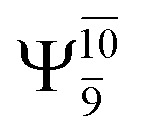	0.9715
	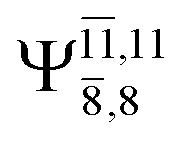	0.0764	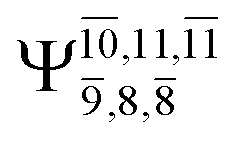	0.0694
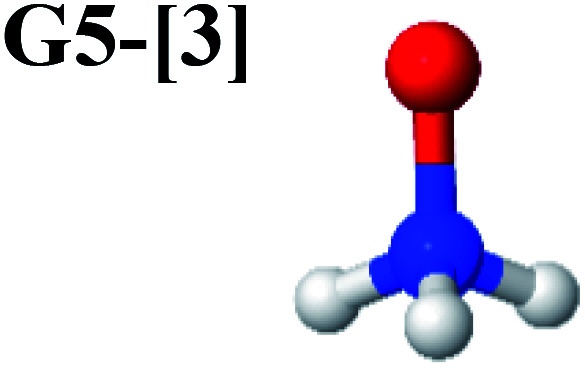	*Ψ*_0_	0.9729	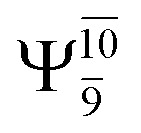	0.9728
	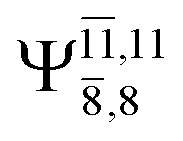	0.0973	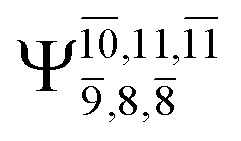	0.1119
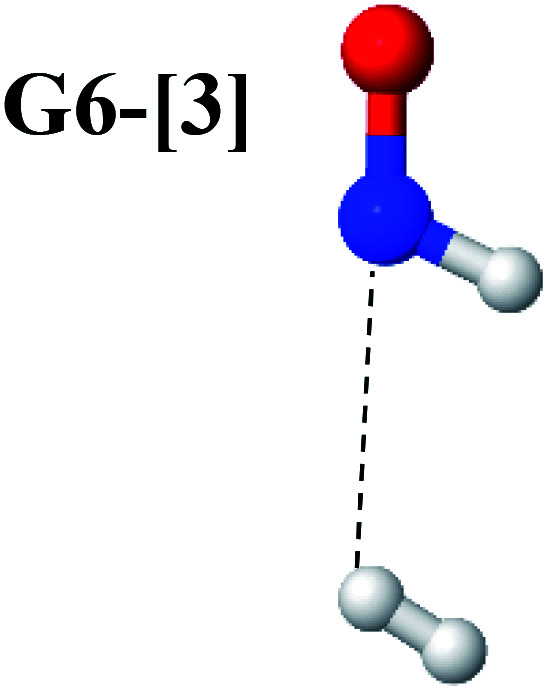	*Ψ*_0_	0.9493	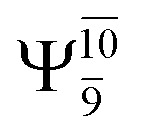	0.9557
	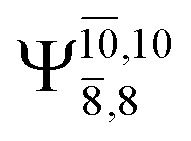	0.1960	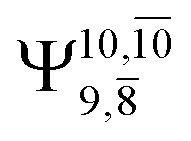	0.0772
	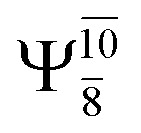	0.0743	—	—
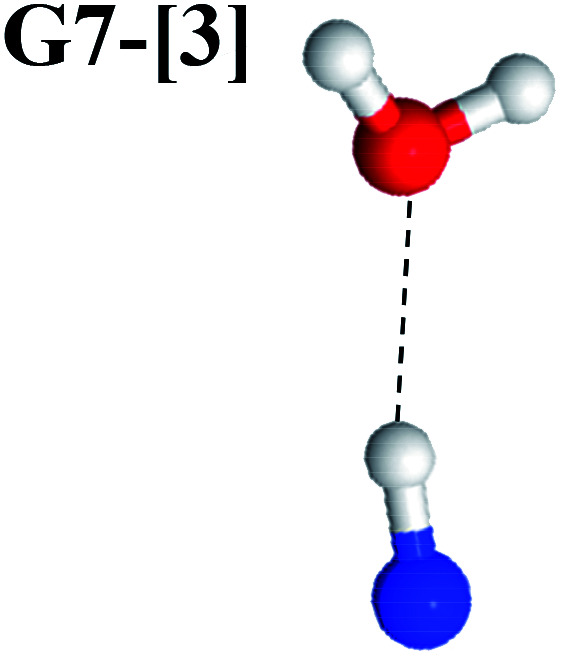	*Ψ*_0_	0.6956	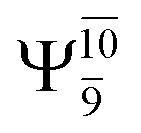	0.9790
	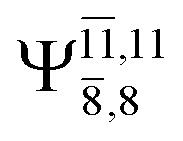	0.0510	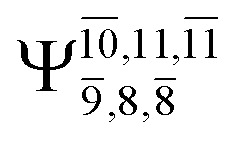	0.0718

### Equilibrium structures

Five equilibrium structures were obtained from the CASPT2(10,9) geometry optimizations in the S_0_ and S_1_ states (Fig. 2). Structure G1-[1] has *C*_s_ symmetry with *R*_N–O_ = 1.47 Å, *R*_O–H_ = 0.97 Å, *R*_N–H_ = 1.03 Å, ∠HNH = 104.6°, ∠HNO = 102.4°, and ∠NOH = 101.4°. These values are in excellent agreement with those obtained from previous *ab initio* calculations^39^ and microwave spectra (*R*_N–O_ = 1.45 Å, *R*_O–H_ = 0.96 Å, *R*_N–H_ = 1.02 Å, ∠HNH = 107.1°, ∠HNO = 103.2°, and ∠NOH = 101.4°).^40^ For structure G1-[1], the CASPT2(10,9) method yields an S_0_ → S_1_ vertical excitation energy (*E*^Ex^) of 6.38 eV (194 nm) with the highest oscillator strength compared with other characteristic structures (Table S12†). These results are in excellent agreement with the photodissociation of NH_2_OH caused by UV absorption at 193 nm (6.42 eV).^5^

Although NH_2_OH is not stable in the S_1_ state and preferentially dissociates into NH_2_O and H, the CASPT2(10,9) geometry was optimized in this state, constraining the O–H distance to its ground state equilibrium value (0.97 Å), because the corresponding structural and energetic data can be used to understand the photodissociation mechanisms. Although the N–H and N–O distances do not change substantially, the three-dimensional (3-D) S_0_ structure G1-[1] is transformed into the planar (2-D) structure E1-[1] of Fig. 2, with a considerably lower vertical excitation energy (*E*^Ex^) of 3.02 eV (411 nm). The change of the NH_2_OH equilibrium structure upon S_0_ → S_1_ excitation (3-D → 2-D) makes it unreasonable to use the freeze-scan method in the construction of the potential energy curves in the excited states.^7^

The CI coefficients of Table 1 evidence that, for structure G1-[1], the electronic ground state, *Ψ*_0_, dominates (C_0_ = 0.9727), with a small contribution from the doubly excited 
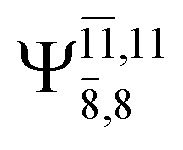
 state 
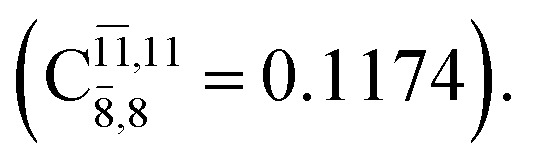
 For comparison, the same structure in the S_1_ state is characterized by a singly excited 
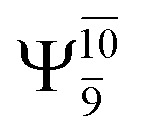
 state 
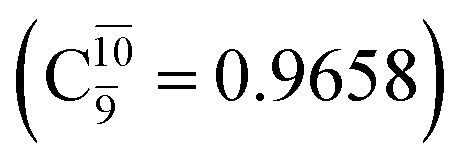
 with a small contribution from the excitation of two electrons of the HOMO−1 orbital (*n*_orb_ = 8), 
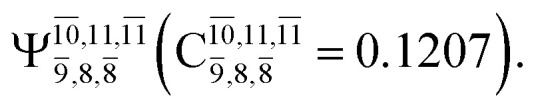
 The interference of the primary electronic states with higher electronic excited states confirms the importance of describing the multiconfigurational character of NH_2_OH. For structure G1-[1], this interference is approximately 12% in both the S_0_ and S_1_ states.

CASPT2(10,9) geometry optimizations reveal that NH_3_O with *C*_3v_ symmetry is stable in both the S_0_ and S_1_ states. The S_0_ state of structure G5-[3], shown in Fig. 2, has *R*_N–O_ = 1.39 Å, *R*_N–H_ = 1.03 Å, ∠HNH = 107.0°, and ∠HNO = 111.8°, in excellent agreement with mass spectroscopic experiments and G2-like *ab initio* calculations (*R*_N–O_ = 1.35 Å, *R*_N–H_ = 1.03 Å, and ∠HNO = 113.7°).^12^ For structure G5-[3], the CASPT2(10,9) method yields a vertical excitation energy (*E*^Ex^) of 4.13 eV (300 nm). The S_0_ → S_1_ excitation leads to structure E5-[3], with small changes in the covalent bond distances and angles: *R*_N–O_ = 1.40 Å, *R*_N–H_ = 1.07 Å, ∠HNH = 109.3°, and ∠HNO = 108.1°. Analysis of the CI coefficients listed in Table 1 shows an electronic state interference similar to the case of G1-[1]: structure G5-[3] is characterized by *Ψ*_0_ (C_0_ = 0.9729), with a small contribution from the doubly excited 
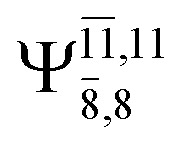
 state 
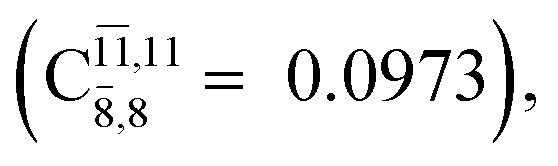
 whereas, in the S_1_ state, 
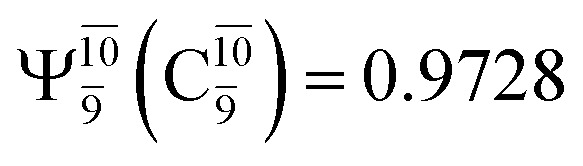
 dominates, with a small contribution from 
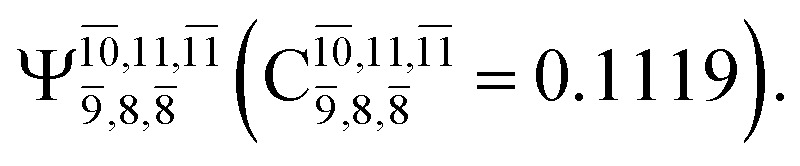


The CASPT2(10,9)-optimized structures of the HNO–H_2_ complex are nearly identical in the S_0_ and S_1_ states, as evidenced by structures G6-[3] and E6-[3] of Fig. 2: in the S_0_ state, *R*_N–O_ = 1.23 Å, *R*_N–H_ = 1.07 Å, and ∠HNO = 107.9°, with a vertical excitation energy (*E*^Ex^) of 1.59 eV (780 nm); in the S_1_ state, *R*_N–O_ = 1.25 Å, *R*_N–H_ = 1.05 Å, and ∠HNO = 115.1°. The equilibrium geometries and *E*^Ex^ are compatible with the results obtained from absorption in the 650–770 nm range (*R*_N–O_ = 1.21 Å, *R*_N–H_ = 1.06 Å, ∠HNO = 109°, and *E*^Ex^ = 1.91–1.61 eV).^18^ The CI coefficients listed in Table 1 reveal that, in the S_0_ state, the electronic ground state *Ψ*_0_ (C_0_ = 0.9493) dominates with ∼21% contributions from the closed-shell excited 
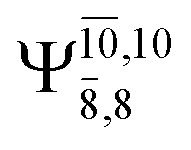
 state 
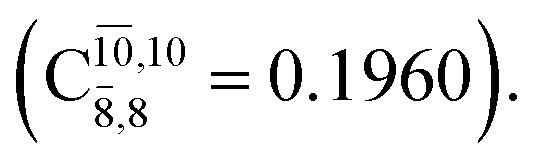
 In the S_1_ state, the same structure is represented by 
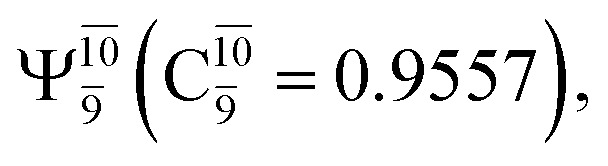
 with a small contribution from 
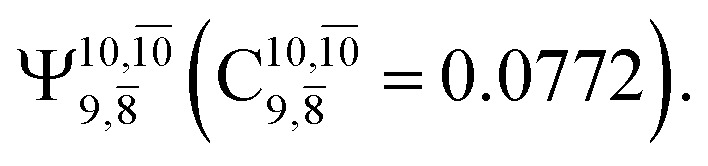
 In this case, the patterns of the CI coefficients differ from those of the previously discussed structures: the primary electronic states interfere with excitations of two electrons from the lone-pair orbital of the O atom (*n*_orb_ = 8) to a dissociated-valence orbital (*n*_orb_ = 11).

Excellent agreement with previous theoretical and experimental data is also found for the NH–H_2_O complex. In this case, the equilibrium geometries obtained from CASPT2(10,9) optimization in the S_0_ and S_1_ states are identical, as shown for structure G7-[3] in Fig. 2, with *R*_N–H_ = 1.04 Å, *R*_O–H_ = 0.97 Å, ∠HOH = 102.9°, and *E*^Ex^ = 0 eV. The patterns of the CI coefficients of structure G7-[3] are the same as those of structures G1-[1], E1-[1], and G5-[3].

Collectively, the structural results, the energetic ones, and the electronic states discussed above confirm the accuracy of the CASPT2(10,9)/aug-cc-pVDZ framework and its applicability to study the photodissociation of NH_2_OH in the S_0_ and S_1_ states.

### O–H dissociation

The O–H dissociation in the S_1_ state is represented by a purely repulsive potential energy curve, as shown in Fig. 3a. Assuming that NH_2_OH completely dissociates into NH_2_O and H in their electronic ground states at the intersection of the S_0_ and S_1_ states (structure E1-[3]^§^, with *E*^Ex^ ≈ 0 eV), the potential energy for the O–H dissociation relative to the vertically excited structure E1-[1]* is Δ*E*^Rel^ = −227 kJ mol^−1^. Instead, in the S_0_ state, the energy barrier (Δ*E*^≠^) with respect to the ground-state equilibrium structure G1-[1] is 386 kJ mol^−1^. The rate constants and relative Gibbs free energies of Tables S8 and S9† confirm that NH_2_OH becomes a photoacid through S_0_ → S_1_ vertical excitation at 194 nm (6.38 eV), and that the nonradiative relaxation of excited NH_2_OH into ground-state NH_2_O and H is thermodynamically favorable: for instance, at 1200 K, Δ*G*^Rel^ = −30 kJ mol^−1^. Oppositely, the thermal dissociation of the O–H bond in the S_0_ state is thermodynamically and kinetically unfavorable: *e.g.*, at 1200 K, Δ*G*^≠^ = 437 kJ mol^−1^ and *k*^Q-vib^ = 2.46 × 10^−6^ s^−1^.

Examination of the S_0_ and S_1_ potential energy curves reveals inflection points at O–H distance *R*_O–H_ = 1.15 Å. Analysis of the CI coefficients of the characteristic structures (Table S1†) shows that, in the S_0_ state, the planar structure with *R*_O–H_ = 0.97 Å is dominated by the electronic ground state *Ψ*_0_ (C_0_ = 0.9789), whereas the singly excited 
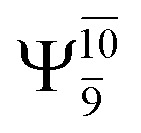
 state 
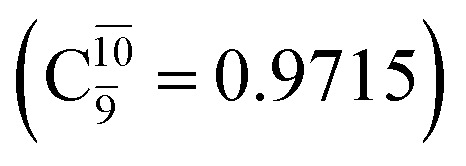
 dominates the S_1_ state. As the O–H distance increases to *R*_O–H_ = 1.15 Å, the electronic states associated with excitations of an electron of the O lone-pair orbital (*n*_orb_ = 8), 
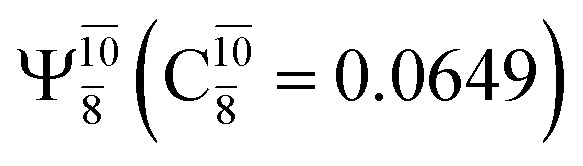
 and 
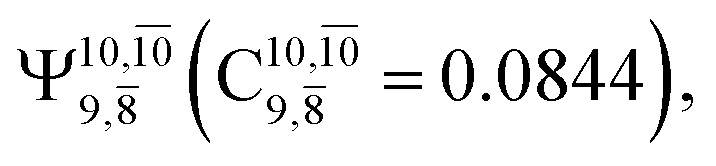
 appear in the S_0_ and S_1_ states, respectively, having their maximum contribution at the intersection of the S_0_ and S_1_ states, 
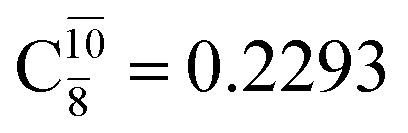
 and 
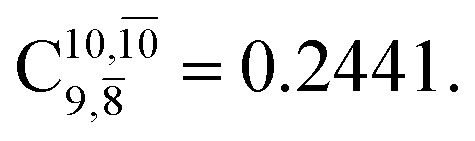
 Therefore, structure E1-[2]^≠^ can be considered as a transition structure and *R*_O–H_ = 1.15 Å as the threshold O–H distance for Rydberg orbital evolution,^7^ beyond which O–H in NH_2_OH dissociates. In this case, the Rydberg orbital (*n*_orb_ = 10) is the natural orbital related to the dissociated H atom. The Gibbs free energy barrier for the Rydberg orbital evolution in the S_0_ state at 1200 K is Δ*G*^≠^ = 210 kJ mol^−1^, with *k*^Q-vib^ = 1.82 × 10^4^ s^−1^ (Table S8†).

It is noteworthy that the conversion of the transition structure into the dissociated products is characterized by significant contributions from excitations of an electron in the lone-pair orbital of the O atom (*n*_orb_ = 8) to the Rydberg orbital (*n*_orb_ = 10). For example, in the S_1_ state, though the contribution of primary electronic state 
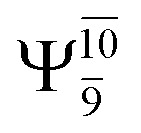
 gradually decreases from 
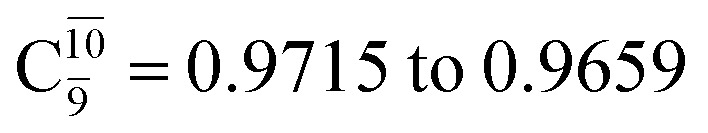
 and 0.9463 for structures E1-[1], E1-[2]^≠^, and E1-[3]^§^, respectively, the contribution of the next excited state, 
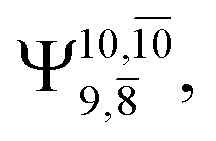
 increases significantly from 
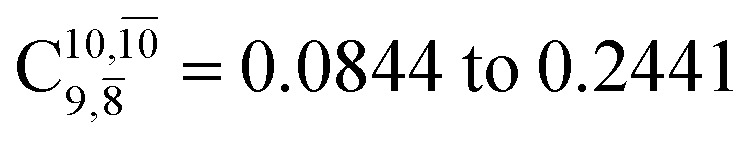
 (nearly 300%) for transition structure E1-[2]^≠^ and product E1-[3]^§^. These values will be used as guidelines to discuss direct covalent bond dissociation and isomerization–dissociation.

### N–O dissociation

Fig. 3b shows the S_1_ potential energy curve obtained from the CASPT2(10,9) calculations. A maximum is seen at *R*_N–O_ = 1.50 Å (structure E2-[2]*) with Δ*E*^≠^ = 11 and Δ*E*^Rel^ = −168 kJ mol^−1^ at the intersection of the S_0_ and S_1_ states, resulting in structure E2-[3]^§^ and G2-[3]^§^ with *R*_N–O_ = 1.90 Å. However, the energy barrier for G2-[3]^§^ formation through N–O dissociation in the S_0_ state is Δ*E*^≠^ = 317 kJ mol^−1^ (Fig. 3b). We recall that the S_1_ potential energy curve for N–O dissociation was calculated by constraining the O–H distance to 0.97 Å because, in the absence of this constraint, the reaction preferentially proceeds towards O–H dissociation. To confirm that structure E2-[2]* is the transition structure for N–O dissociation in the S_1_ state, CASPT2(10,9) geometrical optimizations were performed with no geometrical constraints and starting from slightly shifted *R*_N–O_ values of 1.45 and 1.55 Å. For *R*_N–O_ = 1.45 Å, the results show that structure E2-[2]* relaxes to structure E1-[3]^§^ (the O–H dissociated structure). Instead, for *R*_N–O_ = 1.55 Å, N–O dissociation occurs, yielding NH_2_ and OH (structure E2-[3]^§^).

Overall, these results imply that N–O dissociation cannot proceed directly through the S_0_ → S_1_ vertical excitation of structure G1-[1]. However, the S_0_ and S_1_ potential energy curves shown in Fig. 3b suggest an alternative pathway to avoid the O–H dissociation shown in Fig. 4a. In fact, equilibrium structure G1-[1] in the S_0_ state could be thermally excited and form a precursor in the S_0_ state, *i.e.*, structure G2-[2]^≠^. This structure can be vertically excited to structure E2-[2]* with *E*^Ex^ = 2.85 eV (435 nm), nonradiatively relaxing along a purely repulsive potential energy curve into products NH_2_ and OH in their respective electronic ground states (structure E2-[3]^§^) with Δ*G*^Rel^ = −136 kJ mol^−1^ (Table S9†). Because the N–O dissociation of structure E2-[2]* is barrierless and spontaneous in the S_1_ state, the thermal excitation is the process that determines the rate of generation of structure G2-[2]^≠^; at 1200 K, Δ*G*^≠^ = 195 kJ mol^−1^ and *k*^Q-vib^ = 7.75 × 10^4^ s^−1^ (Table S8†). The photolytic mechanism of the N–O bond at 435 nm is supported by the value of the threshold wavelength that generates NH_2_ and OH from the photoexcitation of NH_2_OH, *λ*_thres_ = 463 nm (2.68 eV).^7,41^

The values of the CI coefficients listed in Table S2† for the N–O dissociation show multiconfigurational trends along the potential energy curves, similar to the O–H dissociation. For example, in the S_1_ state, as the contribution of the primary electronic excited 
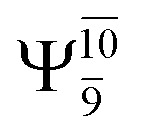
 state gradually changes from 
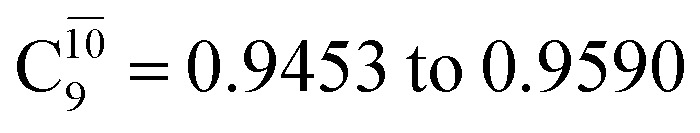
 and 0.9312 for structures E2-[1], E2-[2]*, and E2-[3]^§^, respectively, the contributions arising from excitations of one or two electrons from the HOMO−1 
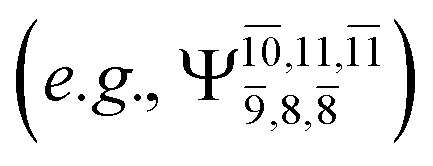
 significantly increase when transition structure E2-[2]* with 
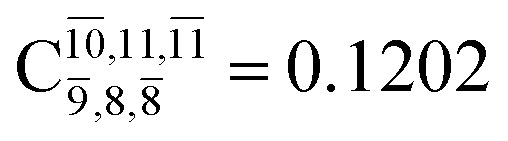
 is converted into structure E2-[3]^§^ (the N–O dissociated structure) with 
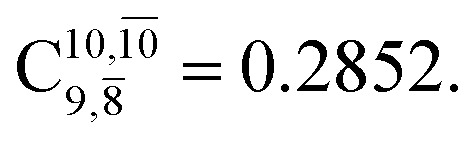
 The finding that structure E2-[2]* (with *R*_N–O_ = 1.50 Å) in the S_1_ state is represented by a slightly longer N–O distance than the equilibrium value (because of Rydberg orbital evolution at *R*_N–O_ slightly longer than 1.47 Å) is consistent with previous *ab initio* calculations using the CASSCF method, which suggested that the Rydberg-valence intersection is close to the N–O equilibrium value.^7^

### N–H dissociation

Two possible N–H dissociation pathways have been considered: N–H(2) and N–H(3), hereafter referred to as N–H^*cis*^ and N–H^*trans*^ dissociations, respectively. Fig. 3c shows that for the N–H^*cis*^ dissociation, the S_1_ potential energy curve with constrained O–H distance (*R*_O–H(5)_ = 0.97 Å) has a maximum at *R*_N–H(2)_ = 1.20 Å (structure E3-[2]*) with Δ*E*^Rel^ = −12 kJ mol^−1^ at the intersection of the S_0_ and S_1_ states, yielding structure E3-[3]^§^ with *R*_N–H(2)_ = 1.45 Å. In the S_0_ state, the energy barrier for the N–H^*cis*^ dissociation (structure G3-[3]^§^) is Δ*E*^≠^ = 378 kJ mol^−1^ (Fig. 3c). For the N–H^*trans*^ dissociation, the S_1_ potential energy curve with constrained O–H distance reveals a maximum at *R*_N–H(3)_ = 1.30 Å (structure E4-[2]*) and Δ*E*^Rel^ = −43 kJ mol^−1^, yielding structure E4-[3]^§^ with *R*_N–H(3)_ = 1.55 Å. Similar to the case of the N–O dissociation, CASPT2(10,9) geometry optimizations confirmed that E3-[2]* and E4-[2]* are the transition structures for the N–H(2) and N–H(3) dissociation pathways, respectively, with threshold N–H distances of 1.20 and 1.30 Å.

In the N–H^*cis*^ dissociation pathway, the S_0_ and S_1_ potential energy curves suggest the possibility to circumvent the O–H dissociation pathway shown in Fig. 4b by thermal excitation of structure G1-[1] to precursor structure G3-[2]^≠^ in the S_0_ state: at 1200 K, Δ*G*^≠^ = 217 kJ mol^−1^ and *k*^Q-vib^ = 8.54 × 10^3^ s^−1^ (Table S8†). Then, structure G3-[2]^≠^ is vertically excited to E3-[2]* with *E*^Ex^ = 2.34 eV (530 nm), and subsequently relaxes into ground-state NHOH and H (structure E3-[3]^§^): at 1200 K, Δ*G*^Rel^ = −63 kJ mol^−1^ (Table S9†). In the N–H^*trans*^ dissociation pathway (Fig. 4b), structure G1-[1] could be thermally excited to structure G4-[2]^≠^ with Δ*G*^≠^ = 286 kJ mol^−1^ and *k*^Q-vib^ = 8.82 × 10^0^ s^−1^ (Table S8†), and the S_0_ → S_1_ vertical excitation with *E*^Ex^ = 1.92 eV (646 nm) leads to structure E4-[2]* and subsequently to ground-state NHOH and H (structure E4-[3]^§^). Because the Δ*G*^≠^ for the formation of the precursor in the S_0_ state (structure G4-[2]^≠^) is rather high even at the highest temperature (1200 K), the N–H^*trans*^ dissociation is thermodynamically unfavorable. Therefore, the N–H^*cis*^ dissociation pathway is preferred, and structure E3-[3]^§^ can be hypothesized as a precursor for the isomerization–dissociation of channels (6) and (7).

Analysis of the main electronic states of the characteristic structures on the potential energy curves (Tables S3 and S4†) for N–H dissociation shows trends of the CI coefficients similar to the case of O–H dissociation. For N–H^*cis*^ dissociation, the electronic ground state *Ψ*_0_ dominates (C_0_ = 0.9807) the S_0_ state, whereas the singly excited 
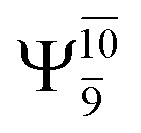
 state 
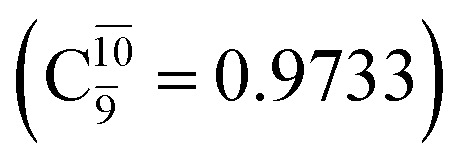
 dominates the S_1_ state. As the N–H(2) distance increases to *R*_N–H(2)_ = 1.20 Å, the electronic states associated with excitations of an electron from the HOMO−1 (*n*_orb_ = 8) to the LUMO (*n*_orb_ = 10), 
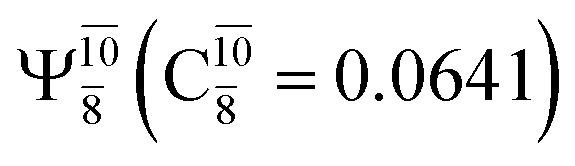
 and 
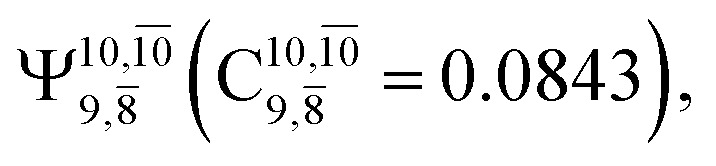
 appear in the S_0_ and S_1_ states, respectively. Their respective maximum, 
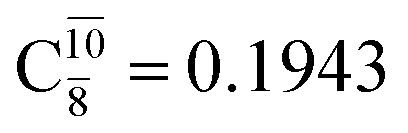
 and 
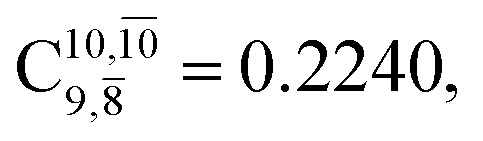
 is observed at the intersection of the S_0_ and S_1_ states, corresponding to dissociated N–H(2). Therefore, E3-[2]^≠^ and *R*_N–H_ = 1.20 Å are confirmed to be the transition structure and the threshold N–H(2) distance for the evolution of the Rydberg orbitals, respectively.

### NH_3_O formation

The relaxed S_1_ potential energy scan suggests that, starting from the O–H dissociated E1-[3]^§^ structure, H(5) → N unimolecular isomerization occurs at *R*_N–H(5)_ = 1.85 Å (structure E5-[1] in Fig. 5a). The results of ref. 11 showed that, in the S_0_ state, the one-step H(5) → N unimolecular isomerization has a high energy barrier (∼234 kJ mol^−1^). In contrast, we found that the formation of NH_3_O from the O–H dissociated structure is barrierless and spontaneous in the S_0_ state (Δ*G*^Rel^ = −205 kJ mol^−1^ at 1200 K, Table S10†), and mediated by transition structure E5-[2]^≠^ (Δ*G*^≠^ = 81 kJ mol^−1^ and *k*^Q-vib^ = 7.55 × 10^9^ s^−1^, Table S11†) in the S_1_ state. Because the total energies of structures E1-[3]^§^ and G5-[1] are similar (Fig. 6a), the two-step pathway involving E1-[3]^§^ as the precursor is the preferred mechanism.

The CI coefficients listed in Table S5† reveal that, in the S_0_ state, the potential energy curve for the H(5) → N isomerization is purely repulsive because of an increase in the contribution of the electronic ground state, *Ψ*_0_: C_0_ = 0.6747, 0.9514, and 0.9715 for structures G5-[1]^§^, G5-[2], and G5-[3], respectively. The increase in C_0_ is accompanied by a significant decrease in the contribution of excitations from the HOMO−1. As an example, 
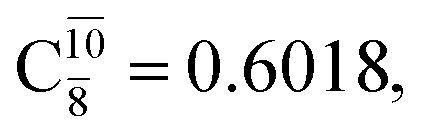

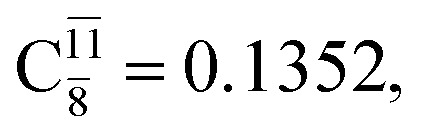
 and 
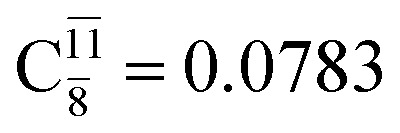
 for G5-[1]^§^, G5-[2], and G5-[3], respectively. In the S_1_ state, the formation of NH_3_O is associated to an increasing energy because of an increase in the contributions of the singly excited 
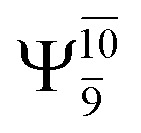
 configuration: 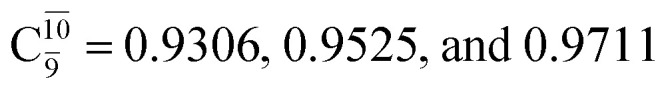 for structures E5-[1], E5-[2]^≠^, and E5-[3], respectively.

### HNO and H_2_ formation

Intuitively, two precursors are possible for isomerization in channel (6), E1-[3]^§^ and E3-[3]^§^, *i.e.*, the O–H(5) and N–H(2) dissociated structure, respectively. Starting from either structure and using *R*_H(2)–H(5)_ = 1.50 Å, H(5) → H(2) isomerization readily occurs at the intersection of the S_0_ and S_1_ states, yielding structure E6-[1]^§^, as shown in Fig. 5b. This suggests that O–H dissociation occurs first. The potential energy curves for the H(5) → H(2) isomerization show two possibilities for the formation of HNO and H_2_, which are structures E6-[3] and G6-[3] in the S_1_ and S_0_ state, respectively. In the S_1_ state, E6-[3] can form with a low free energy barrier *via* transition structure E6-[2]^≠^ (Δ*G*^≠^ = 12 kJ mol^−1^, *k*^Q-vib^ = 7.78 × 10^12^ s^−1^, and Δ*G*^Rel^ = −328 kJ mol^−1^ at 1200 K, Table S11†). The existence of E6-[3] as an equilibrium structure in the S_1_ state supports the experimental finding that the reactive HNO radical has a rather long lifetime (0.1 s) and is one of the dominant products in the gas-phase isolated system.^18^

In contrast, the formation of HNO and H_2_ from structure G6-[1]^§^ is barrierless and spontaneous (Δ*G*^Rel^ = −363 kJ mol^−1^, Table S10†) in the S_0_ state, with structure G6-[3] (*E*^Ex^ = 1.40 eV, corresponding to 886 nm) as the product. The value of *E*^Ex^ is in excellent agreement with the threshold wavelength associated with the formation of HNO and H_2_, *λ*_thres_ = 891 nm (1.39 eV), which was obtained experimentally from the excitation of NH_2_OH by UV photons at 193 nm and thermodynamic data.^5^ The H(5) → H(2) unimolecular isomerization–dissociation mechanisms of the S_0_ and S_1_ states are depicted in Fig. 6b.

The electronic states reported in Table S6† suggest a trend of the CI coefficients for HNO and H_2_ formation similar to that of NH_3_O formation. In the S_0_ state, the reaction is spontaneous because of the increased contributions of *Ψ*_0_ along the potential energy curve (C_0_ = 0.9141, 0.9212, and 0.9493 for structures G6-[1], G6-[2], and G6-[3], respectively). In the S_1_ state, the contribution of 
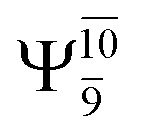
 increases along the S_1_ potential energy curve 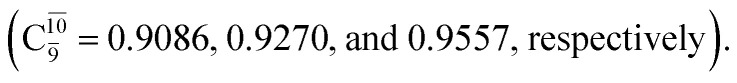


### NH and H_2_O formation

Similar to the HNO and H_2_ case, the potential energy curves for H(2) → O isomerization shown in Fig. 5c evidence the existence of two possibilities for the formation of NH and H_2_O. Starting from the N–H^*cis*^ dissociated structure (structure E3-[3]^§^), the H(2) → O isomerization occurs instantaneously in the S_1_ state (structure E7-[1], *R*_O–H(2)_ = 1.80 Å). As the O–H(2) distance decreases, the S_0_ and S_1_ potential energy curves converge to *R*_O–H(2)_ = 1.30 Å and reach their minimum at *R*_O–H(2)_ = 0.97 Å (structure G7-[3]); at 1200 K, Δ*G*^Rel^ in the S_1_ state is −319 kJ mol^−1^ (Table S11†). The situation is the same in the S_0_ state, in which structure G7-[1] spontaneously turns into structure G7-[3]; at 1200 K, Δ*G*^Rel^ = −152 kJ mol^−1^ (Table S10†). Because the total energies of structures E3-[3]^§^ and G7-[1] are closer than structures E3-[3]^§^ and E7-[1], the H(2) → O unimolecular isomerization–dissociation preferentially occurs in the S_0_ state (Fig. 6c).

For H(2) → O isomerization, the CI coefficients listed in Table S7† show the same multiconfigurational character along the potential energy curves of the H(5) → N and H(2) → H(5) isomerization. In the S_0_ state, the contribution of the electronic ground state increases whereas the contribution of the singly excited state increases in the S_1_ state.

### The interplay between thermal excitations and photoexcitations

The previous sections show in detail the relative Gibbs free energies of the elementary processes and the effects of electronic configuration changes on the potential energy curves. To describe completely the role played by thermal energy in photolytic mechanisms, especially investigating the heat exchange in the endothermic and exothermic processes, the enthalpy changes (Δ*H*) were calculated in the elementary steps. For the direct photolysis of the N–O and N–H covalent bonds, which involves the formation of the precursors in the S_0_ state, the linear relationship between ln *k*^Q-vib^(*T*) and 1/*T* of eqn (9) was used. For the spontaneous isomerization in the S_0_ state (channels (5)–(7), negative Δ*G*^Rel^), the conventional Gibbs free energy change (Δ*G*^Rel^ = Δ*H*^Rel^ − *T*Δ*S*^Rel^) was used to approximate the enthalpies of the exothermic processes (Δ*H*^Rel^). Fig. S2a† shows that the linear relationship between ln *k*^Q-vib^(*T*) and 1/*T* is maintained over the entire temperature range. The values of Δ*H*^≠^ in Table S8† evidence that, for the N–O and N–H^*cis*^ dissociation, the thermal energies required for the formation of the precursors in the S_0_ state are similar to those required for the formation of the Rydberg orbital (structure G1-[2]^≠^), being Δ*H*^≠^ = 190, 208 and 199 kJ mol^−1^ with *k*^Q-vib^(*T*) = 7.75 × 10^4^, 8.54 × 10^3^ s^−1^ and 1.82 × 10^4^, respectively.

For the barrierless, direct covalent bond dissociations in the S_1_ state, the relationship between Δ*G*^Rel^ and *T* is linear over the entire temperature range (Fig. S2b†). Table S9† reveals that the heat release related to O–H and N–H dissociation in the S_1_ state is not substantial, compared with that of N–O dissociation (Δ*H*^Rel^ = −3, −9 and −124 kJ mol^−1^, respectively). Additionally, the exothermic energies of isomerization–dissociation in the S_0_ state (Table S10†) exceed the thermal energy required for the formation of the precursors, Δ*H*^Rel^ = −219 and −279 kJ mol^−1^ for channels (5) and (6), respectively. Assuming that the thermal energies generated in the exothermic processes can be transferred to other NH_2_OH molecules, the exothermic isomerization–dissociation of channel (6), which generates HNO and H_2_, could generate a relevant excess thermal energy for the formation of the precursors in the S_0_ state. Thus, the source of thermal energy required to generate the precursors in the S_0_ state is the formation of HNO and H_2_. This is supported by the finding that the formation of HNO and H_2_ is the preferred process in UV experiments at 193 nm, and that HNO is a dominant product in the gas-phase isolated system.^18^

## Conclusion

The photodissociation mechanisms of NH_2_OH in the lowest singlet-excited state were studied by *ab initio* calculations in the CASPT2(10,9)/aug-cc-pVDZ framework. This study focused on nonradiative relaxation processes that convert the excited-state molecule to its electronic-ground-state products and on the role played by thermal excitation in photodissociation. All the important equilibrium structures in the S_0_ and S_1_ states were characterized, and the potential energy curves for direct covalent bond dissociation and unimolecular isomerization–dissociation were calculated. Additionally, thermodynamic and kinetic data associated with the elementary processes were extracted using the transition state theory.

The CASPT2(10,9) geometry optimizations showed that, in the S_0_ state, the NH_2_OH equilibrium structure is a 3-D structure with *C*_s_ symmetry. An S_0_ → S_1_ vertical excitation energy of 6.38 eV (194 nm) was calculated, and NH_3_O, HNO, and the NH–H_2_O complex were found to be stable in the S_0_ and S_1_ states. Analysis of the CI coefficients of the equilibrium structures revealed that the interference of the primary electronic states with higher excited states is important and that the multiconfigurational character of these structures must be included in *ab initio* studies. Because all the equilibrium structures and energetics are in good agreement with the available theoretical and experimental data, the use of the CASPT2(10,9) method was proved to be appropriate.

The potential energy curves obtained from the CASPT2(10,9) and relaxed scan methods confirmed that O–H dissociation dominates in the S_1_ state. Analysis of the CI coefficients of the characteristic structures on the potential energy curves revealed changes in the multiconfigurational character of the pathway upon O–H dissociation. For example, at the inflection point (*R*_O–H(5)_ = 1.15 Å) of the S_1_ potential energy curve 
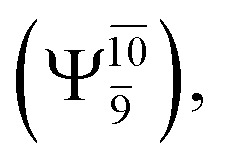
 an electronic state associated with excitation of an electron from the lone-pair orbital of the O atom to the Rydberg orbital 
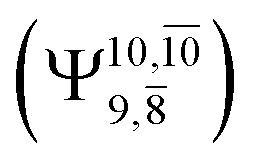
 emerges, having its maximum at the intersection of the S_0_ and S_1_ states. Therefore, the structure at the inflection point is considered a transition structure, and *R*_O–H(5)_ = 1.15 Å is considered to be the threshold distance for the development of Rydberg orbitals, which separates the bound and dissociated electronic states (bound-free transition). These conclusions were used as guidelines to discuss the other photodissociation processes.

Because O–H dissociation is the preferred process in the S_1_ state, the S_1_ potential energy curves for the N–O and N–H dissociations were initially constructed by constraining the O–H distance to its equilibrium S_0_ value. To prevent O–H dissociation, the equilibrium structure in the S_0_ state must be thermally excited to form appropriate precursors, as suggested by the potential energy curves. Then, the thermally excited precursors are vertically excited to form the transition structures in the S_1_ state, which then relax nonradiatively along purely repulsive potential energy curves to generate the products in their respective electronic ground states. Although the required thermal energies are relatively high, according to our thermodynamic and kinetic results, the exothermic energy related to the formation of HNO and H_2_ is at least equally high. Therefore, the thermal excitations in the S_0_ state determine the rate of N–O and N–H dissociation. The proposed mechanisms, which involve different thermally excited precursors, are supported by experimental observations that show that different photon energies lead to different products in their electronic ground state.

The potential energy curves and thermodynamic results revealed that the unimolecular isomerization–dissociation effectively generates products in their electronic ground state through the direct photolysis of the corresponding covalent bonds. In particular, for the formation of HNO and H_2_, the potential energy curves suggested that the high quantum yield of photolysis by UV absorption at 193 nm results from a two-step process: first, the O–H bond dissociates; then, isomerization and the formation of H_2_ in its electronic ground state on a purely repulsive potential curve occur through a strong exothermic process. Overall, the mechanisms proposed in this work emphasize the roles of thermal selectivity and the multiconfigurational character of the associated wavefunctions. Because detailed information on these aspects is limited both theoretically and experimentally, this work provides important insights into the photodissociation of NH_2_OH. Thus, it can be ground for future theoretical and experimental studies of similar systems.

## Conflicts of interest

There are no conflicts to declare.

## Supplementary Material

RA-010-C9RA10956K-s001
