# Supplementary material for: Photolytic mechanisms of hydroxylamine
Source: RSC Adv. 2020 Feb 26;10(14):8319–31. doi: 10.1039/c9ra10956k (PMC9049913; doi:10.1039/c9ra10956k)
Supplement: RA-010-C9RA10956K-s001 [file RA-010-C9RA10956K-s001.pdf]

Electronic Supplementary Material (ESI) for RSC Advances.
This journal is © The Royal Society of Chemistry 2020

Electronic Supplementary Information (ESI)

Photolytic mechanisms of hydroxylamine

by

Jittima Thisuwan

Division of Science Education

Faculty of Education

Nakhon Phanom University,

Nakhon Phanom 48000,

Thailand

Phorntep Promma

and

Kritsana Sagarik*

School of Chemistry

Institute of Science

Suranaree University of Technology

Nakhon Ratchasima 30000

Thailand

Figure S1 a) Schematic diagram showing doubly occupied and active spaces used in CASPT2(10,9) calculations.

b)-c) Spatial distributions of the orbitals potentially involved in $S_0 \rightarrow S_1$ excitation of NH_2OH.

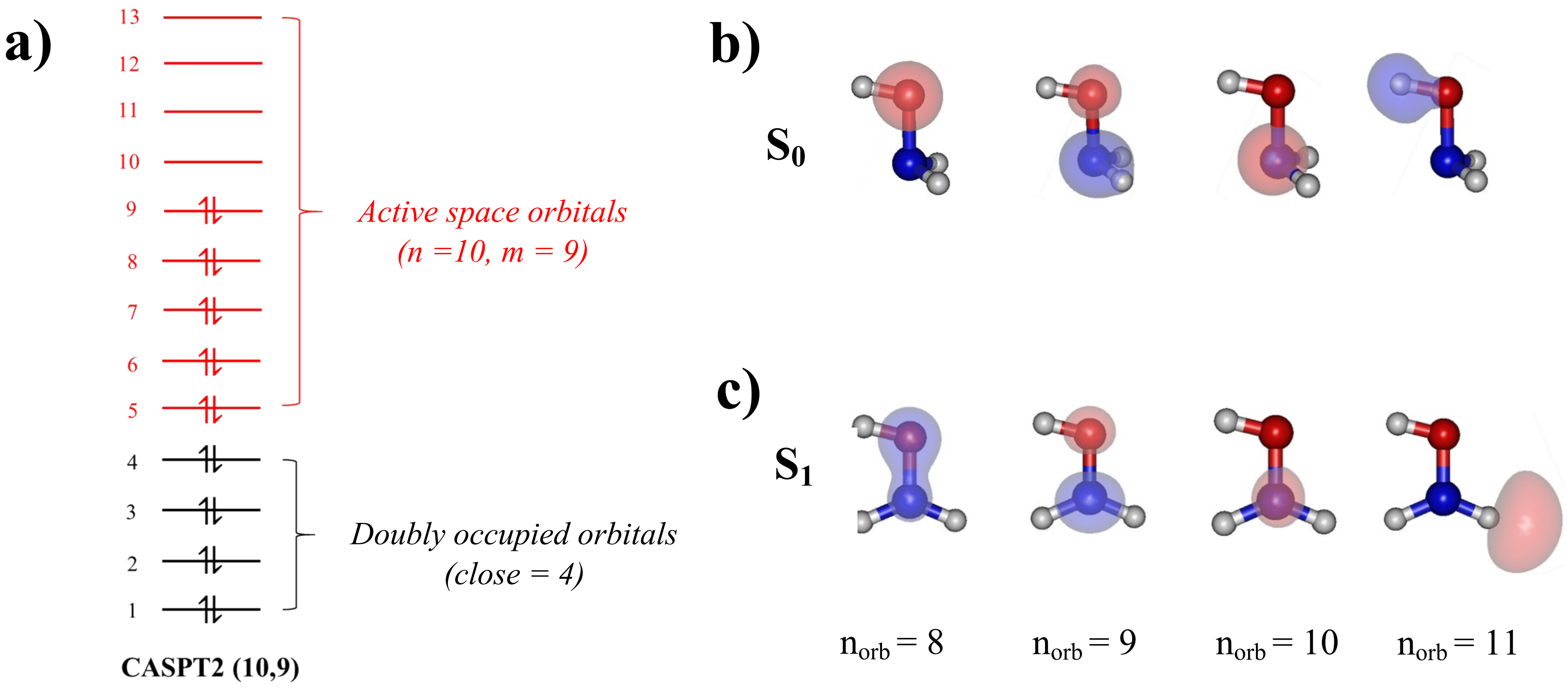

Figure S2 a) Linear relationship between ln $k^{Q\text{-}vib}$ and 1/T used in the calculations of the enthalpy of activation ($\Delta H^{\ddagger}$) for formation of the precursor in the S_0 state (structure **G1-[2]**‡ in channel (1)).

b) Linear relationship between ΔG^{Rel} and T used in the calculation of the enthalpy of the exothermic process (ΔH^{Rel}) in the S_1 state (**E1-[1]**$^{*}\rightarrow$ **E1-[3]**§).

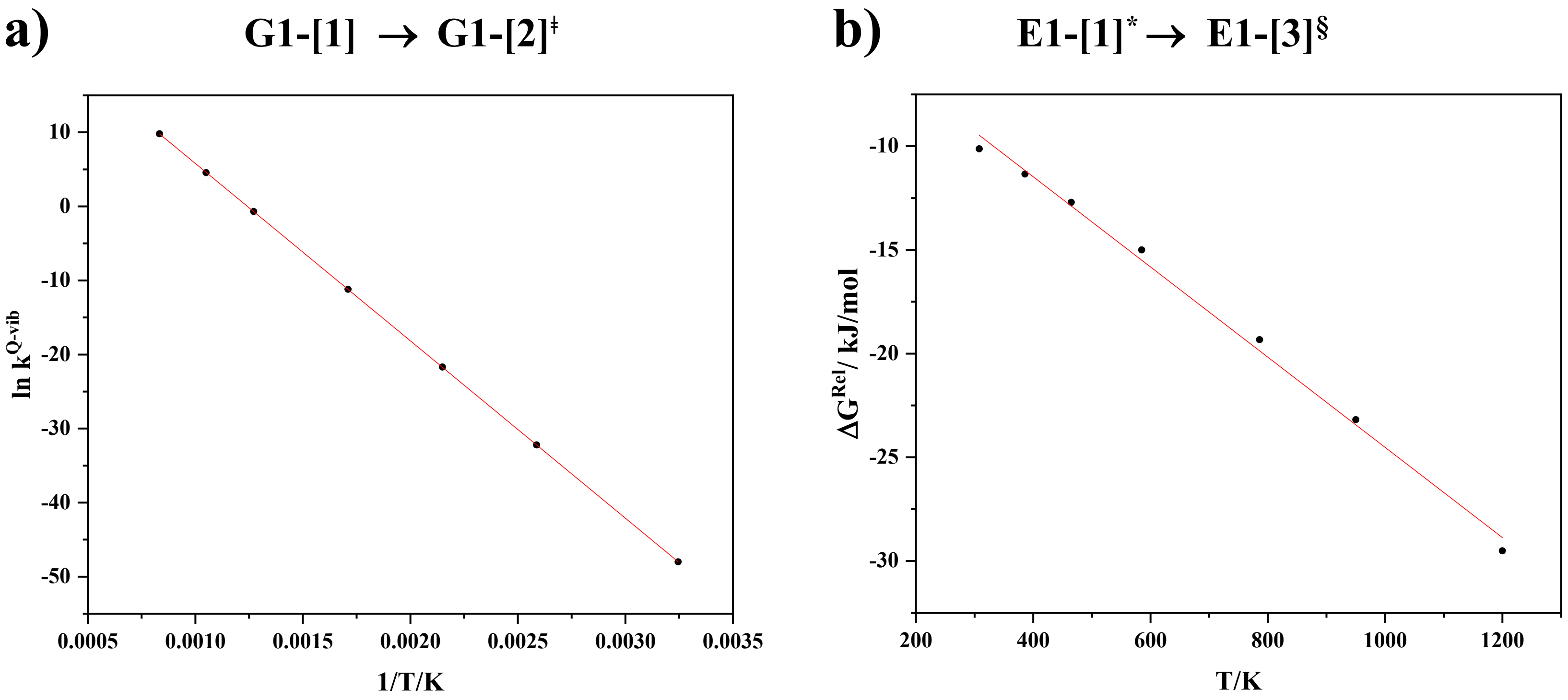

Figure S2a-b

Table S1 Characteristic structures on the S_1 potential energy curve for the O–H dissociation in channel (1). The electronic states and the CI coefficients were

Structures	S_0		S_1	
	Conf.	CI Coeff.	Conf.	CI Coeff.
**E1-[1]** 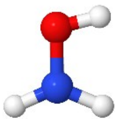	Ψ_0<br>$\Psi_{8,\bar{8}}^{11,\bar{1}1}$	0.9789<br>0.0764	$\Psi_{\bar{9}}^{\bar{1}0}$<br>$\Psi_{9,8,\bar{8}}^{\overline{10},11,\bar{1}1}$	0.9715<br>0.0694
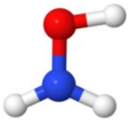	Ψ_0<br>$\Psi_{\bar{8}}^{\bar{1}0}$	0.9730<br>0.0649	$\Psi_{\bar{9}}^{\bar{1}0}$<br>$\Psi_{9,\bar{8}}^{10,\bar{1}0}$	0.9659<br>0.0844
**E1-[3]§** 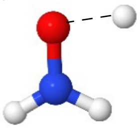	Ψ_0<br>$\Psi_{\bar{8}}^{\bar{1}0}$<br>$\Psi_{8,\bar{8}}^{10,\bar{1}0}$	0.9294<br>0.2293<br>0.2191	$\Psi_{\bar{9}}^{\bar{1}0}$<br>$\Psi_{9,\bar{8}}^{10,\bar{1}0}$<br>-	0.9463<br>0.2441<br>-

obtained from CASPT2(10,9) calculations.

E1-[2]‡

Table S2 Characteristic structures on the S_1 potential energy curve for the N–O dissociation in channel (2). The electronic states and the CI coefficients were obtained from CASPT2(10,9) calculations.

Structures	S$_0$		S$_1$	
	Conf.	CI Coeff.	Conf.	CI Coeff.
**E2-[1]**	Ψ_0<br>$\Psi^{\bar{10},10}_{\bar{8},8}$<br>-	0.9518<br>0.1177<br>-	$\Psi^{\bar{10}}_{\bar{9}}$<br>$\Psi^{10,\bar{11}}_{9,\bar{8}}$<br>$\Psi^{\bar{10},11,\bar{11}}_{\bar{9},8,\bar{8}}$	0.9453<br>0.1591<br>0.0939
**E2-[2]*** 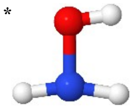	Ψ_0<br>$\Psi^{11,\bar{11}}_{8,\bar{8}}$	0.9700<br>0.1348	$\Psi^{\bar{10}}_{\bar{9}}$<br>$\Psi^{\bar{10},11,\bar{11}}_{\bar{9},8,\bar{8}}$	0.9590<br>0.1202
**E2-[3]§** 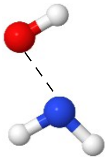	Ψ_0<br>$\Psi^{\bar{10}}_{\bar{8}}$<br>$\Psi^{10,\bar{10}}_{8,\bar{8}}$	0.8839<br>0.3061<br>0.2684	$\Psi^{\bar{10}}_{\bar{9}}$<br>$\Psi^{10,\bar{10}}_{9,\bar{8}}$<br>-	0.9312<br>0.2852<br>-

Table S3 Characteristic structures on the S$_1$ potential energy curve for the N–H^{cis} dissociation in channel (3). The electronic states and the CI coefficients were

Structures	S$_0$		S$_1$	
	Conf.	CI Coeff.	Conf.	CI Coeff.
**E3-[1]**	Ψ_0<br>$\Psi^{11,\bar{11}}_{8,\bar{8}}$	0.9807<br>0.0871	$\Psi^{\bar{10}}_{\bar{9}}$<br>$\Psi^{\bar{10},11,\bar{11}}_{\bar{9},8,\bar{8}}$	0.9733<br>0.0790
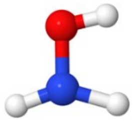	Ψ_0<br>$\Psi^{\bar{10}}_{\bar{8}}$	0.9740<br>0.0641	$\Psi^{\bar{10}}_{\bar{9}}$<br>$\Psi^{10,\bar{10}}_{9,\bar{8}}$	0.9669<br>0.0843
**E3-[3]§** 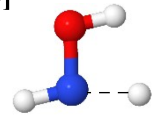	Ψ_0<br>$\Psi^{\bar{10}}_{\bar{8}}$<br>$\Psi^{10,\bar{10}}_{8,\bar{8}}$	0.9438<br>0.1943<br>0.1637	$\Psi^{\bar{10}}_{\bar{9}}$<br>$\Psi^{10,\bar{10}}_{9,\bar{8}}$<br>-	0.9483<br>0.2240<br>-

obtained from CASPT2(10,9) calculations.

E3-[2]*

Table S4 Characteristic structures on the S_1 potential energy curve for the N–H^{trans} dissociation in channel (4). The electronic states and the CI coefficients were obtained from CASPT2(10,9) calculations.

Structures	S_0		S_1	
	Conf.	CI Coeff.	Conf.	CI Coeff.
**E4-[1]**	Ψ_0	0.9790	$\Psi^{\overline{10}}_{\bar{9}}$	0.9716
	$\Psi^{11,\overline{11}}_{8,\bar{8}}$	0.0764	$\Psi^{10,\overline{10}}_{9,\bar{8}}$	0.0753
**E4-[2]*** 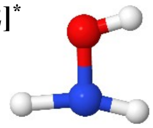	Ψ_0	0.9670	$\Psi^{\overline{10}}_{\bar{9}}$	0.9587
	$\Psi^{11,\overline{11}}_{8,\bar{8}}$	0.1033	$\Psi^{10,\overline{10}}_{9,\bar{8}}$	0.1317
	$\Psi^{10,\overline{10}}_{8,\bar{8}}$	0.0871	-	-
**E4-[3]§** 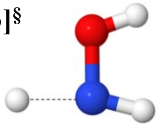	$\Psi^{\overline{10}}_{\bar{9}}$	0.9417	Ψ_0	0.9309
	$\Psi^{10,\overline{10}}_{9,\bar{8}}$	0.2568	$\Psi^{\overline{10}}_{\bar{8}}$	0.2330
	-	-	$\Psi^{10,\overline{10}}_{8,\bar{8}}$	0.1932

Table S5 Characteristic structures on the S_1 potential energy curve for the formation NH_3O in channel (5). The electronic states and the CI coefficients were obtained from CASPT2(10,9) calculations.

Structures	S_0		S_1	
	Conf.	CI Coeff.	Conf.	CI Coeff.
**E5-[1]** 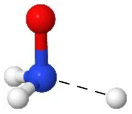	Ψ_0	0.6747	$\Psi_{\bar{9}}^{\overline{10}}$	0.9306
	$\Psi_{\bar{8}}^{\overline{10}}$	0.6018	$\Psi_{9,8}^{10,\overline{10}}$	0.2924
	$\Psi_{8,\bar{8}}^{10,\overline{10}}$	0.3501	-	-
**E5-[2]‡** 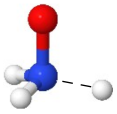	Ψ_0	0.9514	$\Psi_{\bar{9}}^{\overline{10}}$	0.9525
	$\Psi_{\bar{8}}^{\overline{11}}$	0.1352	$\Psi_{9,8}^{10,\overline{11}}$	0.1129
	$\Psi_{8,\bar{8}}^{11,\overline{11}}$	0.0952	$\Psi_{\bar{9},8,\bar{8}}^{\overline{10},11,\overline{11}}$	0.1071
**E5-[3]** 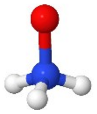	Ψ_0	0.9715	$\Psi_{\bar{9}}^{\overline{10}}$	0.9711
	$\Psi_{8,\bar{8}}^{11,\overline{11}}$	0.1018	$\Psi_{\bar{9},8,\bar{8}}^{\overline{10},11,\overline{11}}$	0.1180
	$\Psi_{\bar{8}}^{\overline{11}}$	0.0783	-	-

Table S6 Characteristic structures on the S_1 potential energy curve for the formation of HNO and H_2 in channel (6). The electronic states and the CI coefficients were obtained from CASPT2(10,9) calculations.

<table>
<tr><th rowspan="2">Structures</th><th colspan="2">S_0</th><th colspan="2">S_1</th></tr>
<tr><th>Conf.</th><th>CI Coeff.</th><th>Conf.</th><th>CI Coeff.</th></tr>
<tr><td>E6-[1] 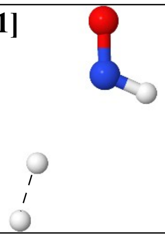</td><td>Ψ_0
$\Psi^{10,\overline{10}}_{8,\overline{8}}$</td><td>0.9141
0.2000</td><td>$\Psi^{\overline{10}}_{\overline{9}}$
$\Psi^{10,\overline{11}}_{9,\overline{9}}$</td><td>0.9086
0.2675</td></tr>
<tr><td>E6-[2]‡ 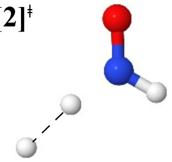</td><td>Ψ_0
$\Psi^{\overline{10}}_{\overline{7}}$</td><td>0.9212
0.0628</td><td>$\Psi^{\overline{10}}_{\overline{9}}$
$\Psi^{\overline{10},11,\overline{11}}_{9,8,\overline{8}}$</td><td>0.9270
0.2569</td></tr>
<tr><td>E6-[3] 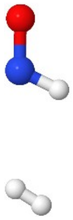</td><td>Ψ_0
$\Psi^{10,\overline{10}}_{8,\overline{8}}$
$\Psi^{\overline{10}}_{\overline{8}}$</td><td>0.9493
0.1960
0.0743</td><td>$\Psi^{\overline{10}}_{\overline{9}}$
$\Psi^{10,\overline{10}}_{9,\overline{8}}$
-</td><td>0.9557
0.0772
-</td></tr>
</table>

Table S7 Characteristic structures on the S_1 potential energy curve for the formation of NH-H_2O complex. The electronic states and the CI coefficients were obtained from CASPT2(10,9) calculations.

Structures	S$_0$		S$_1$	
	Conf.	CI Coeff.	Conf.	CI Coeff.
**E7-[1]** 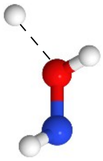	$\Psi_{\bar{9}}^{\bar{1}0}$<br>$\Psi_{9,\bar{8}}^{10,\bar{1}0}$<br>-	0.9310<br>0.2992<br>-	Ψ_0<br>$\Psi_{\bar{8}}^{\bar{1}0}$<br>$\Psi_{8,\bar{8}}^{10,\bar{1}0}$	0.6965<br>0.5798<br>0.3567
**E7-[2]§** 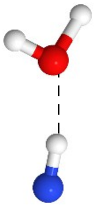	Ψ_0<br>$\Psi_{\bar{8},8}^{\overline{11},11}$	0.6858<br>0.1057	$\Psi_{\bar{9}}^{\bar{1}0}$<br>$\Psi_{\bar{9},8,\bar{8}}^{\overline{10},11,\bar{1}1}$	0.9706<br>0.1496
**G7-[3]** 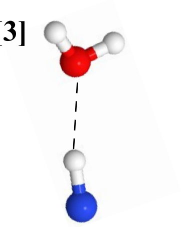	Ψ_0<br>$\Psi_{\bar{8},\,8}^{\bar{1}1,11}$	0.6956<br>0.0510	$\Psi_{\bar{9}}^{\bar{1}0}$<br>$\Psi_{\bar{9},8,\bar{8}}^{\overline{10},11,\bar{1}1}$	0.9790<br>0.0718

Table S8 Rate constants (k), enthalpies of activation ($\Delta H^‡$) and Gibbs free energy barriers ($\Delta G^‡$) for the formation of the precursors in the direct covalent bond dissociations in the S_0 state, obtained based on transition state theory and the potential energy curves. Energies, rate constants and temperatures are in kJ mol^{-1}, s^{-1} and K, respectively.

	**$\Delta E^‡$**	**$\Delta H^‡$**	**T_c**	**T**	**k^{Class}**	**$k^{Q\text{-}vib}$**	**$k^{S\text{-}Wig}$**	**$\Delta G^‡$**
				308	1.07×10^{-22}	1.42×10^{-21}	4.81×10^{-21}	199
**G1-[1] → G1-[2]‡**	209	199	371	585	4.00×10^{-6}	1.38×10^{-5}	2.29×10^{-5}	201
				1200	1.18×10^{4}	1.82×10^{4}	2.11×10^{4}	210
				308	3.42×10^{-19}	8.42×10^{-20}	1.81×10^{-19}	188
**G1-[1] → G2-[2]‡**	216	190	257	585	2.53×10^{-5}	1.38×10^{-4}	1.82×10^{-4}	190
				1200	4.46×10^{4}	7.75×10^{4}	8.34×10^{4}	195
				308	3.72×10^{-24}	4.75×10^{-23}	9.21×10^{-23}	207
**G1-[1] → G3-[2]‡**	222	208	232	585	7.49×10^{-7}	2.39×10^{-6}	3.01×10^{-6}	209
				1200	5.79×10^{3}	8.54×10^{3}	9.07×10^{3}	217
				308	1.37×10^{-37}	3.00×10^{-35}	8.02×10^{-35}	279
**G1-[1] → G4-[2]‡**	314	281	310	585	9.87×10^{-14}	9.87×10^{-13}	1.44×10^{-12}	281
				1200	4.24×10^{0}	8.82×10^{0}	9.80×10^{0}	286

$\Delta E^‡$ = energy barrier with respect to structure **G1-[1]**; T_c = crossover temperature; ‡ = transition structure; k^{Class} = rate constant obtained from classical transition state theory (TST); $k^{Q\text{-}vib}$ = rate constant obtained with quantized vibrations including the zero-point vibrational energy; $k^{S\text{-}Wig}$ = rate constant obtained with quantized vibrations and tunneling correction through the simplified Wigner correction to the second order; $\Delta H^‡$ and $\Delta G^‡$ = enthalpy of activation and Gibbs free energy barrier with respect to structure **G1-[1]**.

Table S9 Enthalpies (ΔH^{Rel}) and relative Gibbs free energies (ΔG^{Rel}) for direct covalent bond dissociations in the S_1 state, obtained based on the transition state theory and barrierless potential energy curves ($\Delta E^{‡} = 0$ kJ mol^{-1}). Energies and temperatures are in kJ mol^{-1} and K, respectively.

	**ΔE^{Rel}**	**ΔH^{Rel}**	**T**	**ΔG^{Rel}**
**E1-[1]* → E1-[3]§**	-227	-3	308	-10
			585	-15
			1200	-30
**E2-[2]* → E2-[3]§**	-168	-124	308	-127
			585	-130
			1200	-136
**E3-[2]* → E3-[3]§**	-12	-9	308	-12
			585	-28
			1200	-63

ΔE^{Rel} = relative energy with respect to the vertically excited structure; * = vertically excited structure; § = structure at the intersection of the S_0 and S_1 potential energy curves; ΔH^{Rel} and ΔG^{Rel} = enthalpy and relative Gibbs free energy with respect to the vertically excited structure.

Table S10 Enthalpies (ΔH^{Rel}) and relative Gibbs free energies (ΔG^{Rel}) for formations of NH_3O, HNO and H_2, and the N–H..H_2O complex obtained from unimolecular isomerizations of dissociated structures in the S_0 state,

computed based on transition state theory and barrierless potential energy curves ($\Delta E^{\ddagger} = 0$ kJ mol^{-1}). Energies and temperatures are in kJ mol^{-1} and K, respectively.

	ΔE^{Rel}	ΔH^{Rel}	T	ΔG^{Rel}
			308	-215
**G5-[1]§ → G5-[3]**	-241	-219	585	-212
			1200	-205
			308	-301
**G6-[1]§ → G6-[3]**	-285	-279	585	-319
			1200	-363
			308	-154
**G7-[1] → G7-[3]**	-180	-154	585	-152
			1200	-152

ΔE^{Rel} = relative energy with respect to the precursor; § = structure at the intersection of the S_0 and S_1 potential energy curves; ΔH^{Rel} and ΔG^{Rel} = enthalpy and relative Gibbs free energy with respect to dissociated structure.

Table S11 Rate constants (k), enthalpies (ΔH) and Gibbs free energies (ΔG) for formations of NH_3O, HNO and H_2, and the N-H..H_2O complex from unimolecular isomerizations of dissociated structures in the S_1 state, obtained based on the transition state theory and potential energy curves. Energies, rate constants and temperatures are in kJ mol^{-1}, s^{-1} and K, respectively.

	$\Delta E^{‡ (Rel)}$	$\Delta H^{‡ (Rel)}$	T_c	T	k^{Class}	$k^{Q\text{-}vib}$	$k^{S\text{-}Wig}$	$\Delta G^{‡ (Rel)}$
				308	7.98×10^{2}	2.40×10^{3}	1.04×10^{4}	56
**E5-[1] → E5-[2]‡**	55	52	437	585	2.01×10^{7}	3.14×10^{7}	6.03×10^{7}	63
				1200	6.56×10^{9}	7.55×10^{9}	9.20×10^{9}	81
				308				(-9)
**E5-[2]‡ → E5-[3]**	(-41)	(-12)	-	585	-	Barierless potential	-	(-7)
				1200				(-1)
				308	6.38×10^{3}	5.04×10^{5}	1.42×10^{6}	42
**E6-[1]§ → E6-[2]‡**	71	57	324	585	2.87×10^{9}	1.49×10^{10}	2.24×10^{10}	33
				1200	4.88×10^{12}	7.78×10^{12}	8.71×10^{12}	12
				308				(-234)
**E6-[2]‡ → E6-[3]**	(-221)	(-201)	-	585	-	Barierless potential	-	(-262)
				1200				(-328)
				308				(-254)
**E7-[1] → E7-[3]§**	(-249)	(-230)	-	585	-	Barierless potential	-	(-272)
				1200				(-319)

$\Delta E^{‡}$ and ΔE^{Rel} = relative energies on the potential energy curve with respect to the precursor; T_c = crossover temperature; ‡ = transition structure; § = structure at the intersection of the S_0 and S_1 potential energy curves; k^{Class} = rate constant obtained from classical transition-state theory (TST); $k^{Q\text{-}vib}$ = rate constant obtained with quantized vibrations including the zero-point vibrational energy; $k^{S\text{-}Wig}$ = rate constant obtained with quantized vibrations and tunneling correction through the simplified Wigner correction to the second order; $\Delta H^{‡}$ and $\Delta G^{‡}$ = enthalpy and Gibbs free energy barrier with respect to the precursor; ΔH^{Rel} and ΔG^{Rel} = enthalpy and relative Gibbs free energy with respect to the transition structure; (…) = value obtained based on barrierless potential.

Table S12 Vertical excitation wavelengths (λ) and energies (E^{Ex}) and corresponding normalized oscillator strengths with respect to structure **G1-[1]**.

**Structure**	**λ (nm)**	**E^{Ex} (eV)**	**Oscillator strength**
**G1-[1]**	194	6.38	1.00
**G2-[2]**‡	435	2.85	0.007
**G3-[2]**‡	530	2.34	0.010
**G4-[2]**‡	646	1.92	0.010
